# Lost in translation: conserved amino acid usage despite extreme codon bias in foraminifera

**DOI:** 10.1128/mbio.03916-24

**Published:** 2025-03-05

**Authors:** Auden E. Cote-L'Heureux, Elinor G. Sterner, Xyrus X. Maurer-Alcalá, Laura A. Katz

**Affiliations:** 1 Department of Biological Sciences, Smith College, Northampton, Massachusetts, USA; 2 Division of Invertebrate Zoology, Sackler Institute for Comparative Genomics, American Museum of Natural History 5963 , New York, New York, USA; 3 Program in Organismic Biology and Evolution, University of Massachusetts Amherst, Amherst, Massachusetts, USA; University of California Los Angeles, Los Angeles, CA, USA

**Keywords:** foraminifera, codon usage, compositional bias, amino acid usage, GC content

## Abstract

Analyses of codon usage in eukaryotes suggest that amino acid usage responds to GC pressure so AT-biased substitutions drive higher usage of amino acids with AT-ending codons. Here, we combine single-cell transcriptomics and phylogenomics to explore codon usage patterns in foraminifera, a diverse and ancient clade of predominantly uncultivable microeukaryotes. We curate data from 1,044 gene families in 49 individuals representing 28 genera, generating perhaps the largest existing dataset of data from a predominantly uncultivable clade of protists, to analyze compositional bias and codon usage. We find extreme variation in composition, with a median GC content at fourfold degenerate silent sites below 3% in some species and above 75% in others. The most AT-biased species are distributed among diverse non-monophyletic lineages. Surprisingly, despite the extreme variation in compositional bias, amino acid usage is highly conserved across all foraminifera. By analyzing nucleotide, codon, and amino acid composition within this diverse clade of amoeboid eukaryotes, we expand our knowledge of patterns of genome evolution across the eukaryotic tree of life.

**IMPORTANCE:**

Patterns of molecular evolution in protein-coding genes reflect trade-offs between substitution biases and selection on both codon and amino acid usage. Most analyses of these factors in microbial eukaryotes focus on model species such as *Acanthamoeba, Plasmodium,* and yeast, where substitution bias is a primary contributor to patterns of amino acid usage. Foraminifera, an ancient clade of single-celled eukaryotes, present a conundrum, as we find highly conserved amino acid usage underlain by divergent nucleotide composition, including extreme AT-bias at silent sites among multiple non-sister lineages. We speculate that these paradoxical patterns are enabled by the dynamic genome structure of foraminifera, whose life cycles can include genome endoreplication and chromatin extrusion.

## INTRODUCTION

The “universal” genetic code comprises 61 codons that encode 20 amino acids, resulting in redundancy whereby most amino acids are encoded by two or more codons. In many organisms, the use of these redundant codons is not equal (e.g., references 1 and 2). Nucleotide composition across most of the genome (e.g., intergenic regions, repetitive elements) is driven in part by mutational dynamics plus processes like biased gene conversion, and in part by selection (3–5). Within protein-coding regions, bias in nucleotide substitutions is mitigated by functional constraints on amino acid sequences, which is largely reflected in the first and second positions of codons. Here we expand the understanding of how principles of genome evolution vary across the eukaryotic tree of life by describing patterns of nucleotide, codon, and amino acid composition in foraminifera, an ancient and diverse clade of microbial eukaryotes. Previous analyses of microbial lineages have expanded our knowledge of genome evolution, as in the discoveries of telomeres (ciliates), self-splicing RNAs (ciliates), and RNA editing (e.g., trypanosomes [6]).

While genome and protein composition have been investigated in a large number of bacterial species (7–9), in eukaryotes data come largely from studies that have focused on “model” lineages (1, 2, 10). Trade-offs between substitution bias and selection have been argued to shape codon usage in lineages such as *Drosophila* (11), the fungi *Saccharomyces cerevisiae* (12) and *Neurospora* (13), the parasites *Plasmodium* (14)*, Schistosoma* (15) and *Giardia* (16), and the amoeba *Dictyostelium* (5). One “rule” that has emerged from past studies is that taxa with biased composition tend to show corresponding bias in amino acid usage as a result of “GC pressure,” despite constraints on protein function (17, 18). For example, the AT-rich *Plasmodium falciparum* favors amino acids with AT-rich codons (i.e., “FYMINK”) while the GC-rich *Acanthamoeba* uses a greater proportion of amino acids with GC-rich codons (“GARP”) (19).

While the uneven usage of synonymous codons often arises from neutral processes such as background substitution bias, selection can also play a role. Selection for optimal codon usage may reflect a drive toward more efficient translation through changes in mRNA structure, or a pressure to use codons and/or amino acids whose tRNAs have greater copy number and are therefore more readily available (20–23). However, direct measurements of tRNA abundance are scarce, and this hypothesis fails to account for other dynamics of translation (e.g., initiation as the rate-limiting step [20, 22, 23]). Selection for translational accuracy may also drive synonymous codon usage bias, with greater pressure to use codons less prone to mistranslation at sites key to a protein’s function (24, 25). The mutation-selection-drift model predicts that drift and mutational processes are reflected in the composition of lowly expressed genes, while highly expressed genes are more exposed to selective pressures (26) (but see reference 27). Hence, comparisons between highly and lowly expressed genes can help to disentangle the effects of mutational and selective forces in driving patterns of codon and amino acid usage (28, 29).

Studies of microbial eukaryotes often use data from only a limited number of species or find relatively little variation between species (e.g., references 30–33). Genome composition has been well studied in some parasitic microeukaryotes, yielding some novel evolutionary insights, for example into the role of codon usage in the host range of parasitic fungi (34) and in driving patterns in co-transcribed genes in *Trypanosoma* (35). Ciliates have been shown to often use non-canonical genetic codes (i.e., with re-assigned stop codons), yielding interesting insights into fundamental features of eukaryotic genomes (36, 37). Recent work in diatoms and other phytoplankton has shown the potential of sampling thoroughly from a group of microeukaryotes to reveal emergent trends in patterns of molecular evolution (38, 39), but this has yet to be replicated in other clades of unicellular eukaryotes.

Foraminifera, the focus of this study, are an ancient, species-rich (~10,000 species [40]) clade of microeukaryotes that are characterized by the presence of a test (i.e., shell, though this has been lost in a few lineages) and fine, anastomosing pseudopodia (40, 41). Because of their robust fossil record and large sizes (up to 10 cm), these single-celled organisms have a rich history of study (reviewed in reference 40). The current phylogeny of Foraminifera shows the single-chambered “monothalamids” as a non-monophyletic group that includes the early-diverging allogromids, with two major transitions into multi-chambered groups: the Globothamea and Tubothalamea (41, 42). Very little is known about the structure of foraminiferan genomes as a highly contaminated first genome assembly has yielded few insights (43). Moreover, only a limited number of transcriptomes are available for foraminifera, and many of these are highly contaminated with symbionts and environmental sequences (42).

Here, we analyze transcriptomic data from 49 foraminifera (28 generated in our lab, 21 from GenBank; File S1 available through Figshare at https://doi.org/10.6084/m9.figshare.28083020) covering 28 foraminiferal genera. We extensively curate these data to mitigate the effects of contamination, focusing on 1,044 gene families as seeded by OrthoMCL (44) and processed by our phylogenomic pipeline PhyloToL (45). To interpret patterns of molecular evolution across protein genes, we concatenated 171 of these gene families and constructed a phylogeny that is largely consistent with prior studies (41, 42, 46–48). We then analyzed the composition bias and codon usage of the 1,044 gene families to document a wide range in silent-site GC content coupled with conserved amino acid usage. We use a number of metrics to describe these trends and discuss the potential underlying forces driving the extreme patterns of codon usage coupled with conserved amino acid usage.

## RESULTS AND DISCUSSION

**Fig 1:**
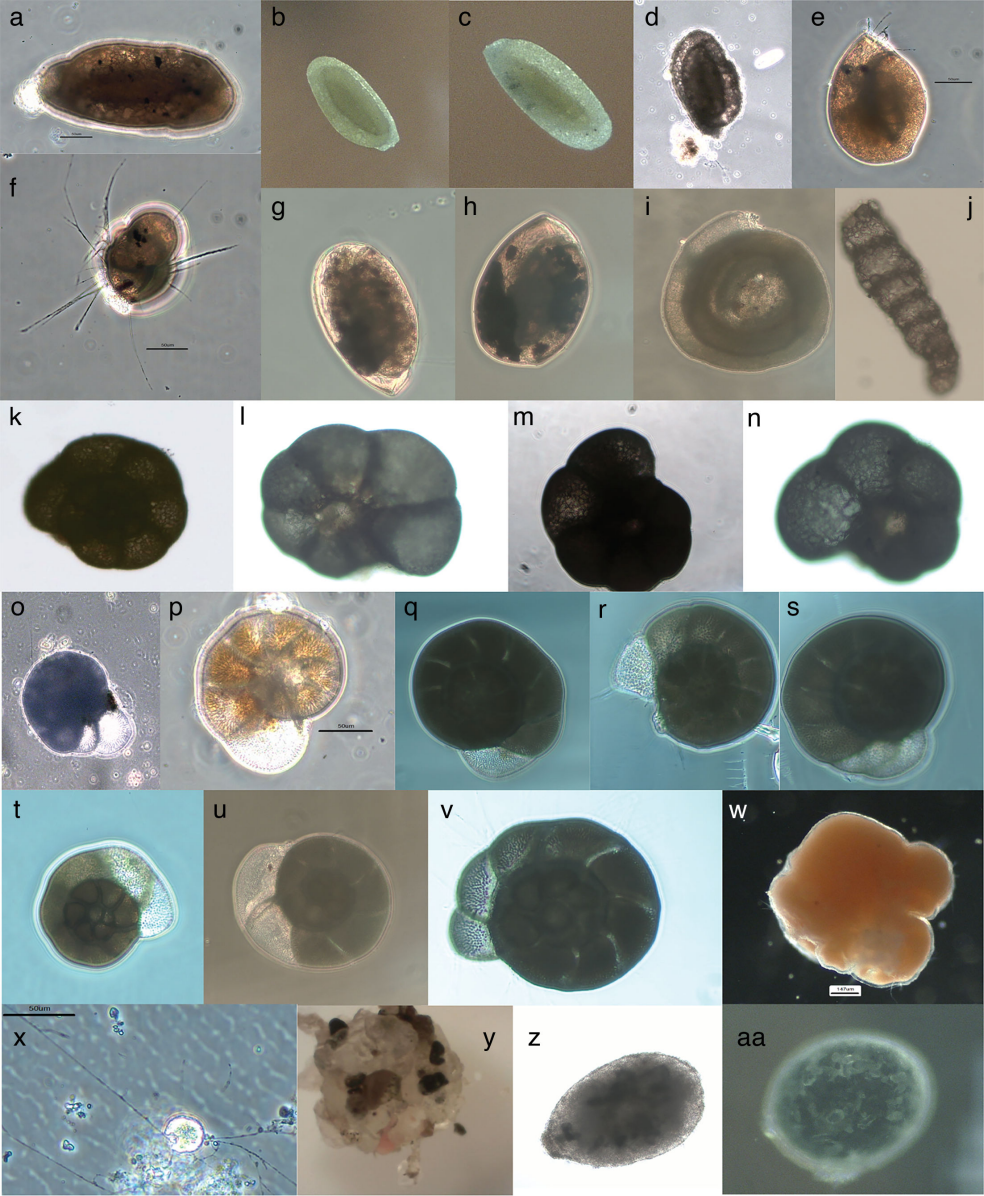
Images of foraminifera isolated in the Katz lab. Cells are organized roughly taxonomically and with all but three (labeled “exemplar”) pictures of individuals isolated for transcriptomes. Tubothalamids: (a) *Miliammina-*like sp. 1; (b, c) *Miliammina* sp. 1; (d) *Miliammina* sp. 2; (e, f) *Triloculina* sp.; (g, h) *Quinqueloculina* sp.; (i) *Ammodiscus* sp. Globothalamids: (j) *Reophax*-like; (k) a *Trochammina* test that we infer contained a *Vellaria*-like monothalamid squatter (see text); (l, m) *Trochammina*-like sp. 1; (n) *Trochammina*-like sp. 2; (o) *Haynesina*-like; (p) exemplar image of *Haynesina orbiculare*; (q–s) *Ammonia* sp. 1; (t, u) *Ammonia* sp. 2; (v) *Ammonia* sp. 3. Monothalamids: (w) exemplar image of *Allogromia arnoldi*; (x) freshwater foram; (y) *Notodendrodes hyalinosphaira*; (z-aa) *Psammophaga* sp. Note that these images were taken efficiently ahead of RNA isolation for transcriptomics, and they are intended only to provide an overview of test morphology; all cells showed active pseudopods at the time of isolation.

To explore the evolution of compositional bias and both codon and amino acid usage among foraminifera, we analyzed single-cell transcriptome data using PhyloToL (45) plus a set of custom scripts (https://github.com/AudenCote/ForamCodonUsage). The full data set includes 49 cells, 28 isolated in our lab (Fig. 1), and the others from GenBank, which represent 28 genera distributed among Tubothalamea, Globothalamea (Textulariida and Rotaliida), and Xenophyophorea plus non-monophyletic monothalamids (Table 1; File S1 available through Figshare at https://doi.org/10.6084/m9.figshare.28083020). We analyze patterns within foraminifera and then compare the resulting insights to the literature. We aim to expand our understanding of the patterns and processes underlying molecular evolution in a diverse clade of unicellular eukaryotes and provide a precedent for future analyses of other uncultivable lineages.

**TABLE 1:** Summary of data included in this study, with genera with extreme bias indicated in bold^*a*^

Clade/category	No. of genera	No. of cells	Genera
Monothalamids	8	10	***Allogromia****, Hippocrepina, Hippocrepinella, Notodendrodes, Psammophaga, Toxisarcon, Vellaria*-like*,* freshwater foram
Globothalamea	12	26	*Ammonia, Amphistegina, **Bolivina**, Cribrostomoides, Eggerelloides, Elphidium, Haynesina*, *Nonionella*-like, *Reophax*-like, ***Spiroplectammina***, *Textularia*, ***Trochamminia*-like**
Tubothalamea	5	10	***Ammodiscus, Miliammina, Quinqueloculina, Sorites, Triloculina***
Xenophyophorea	3	3	*Aschemonella, Psammina, Shinkaiya*

^*a*^ See Fig. 1 for images and File S1, available through Figshare at https://doi.org/10.6084/m9.figshare.28083020, for more details on the identification and naming of each cell.

### Curation of transcriptomic data for analyses

We provide a substantial expansion of ‘omics data for foraminifera, curating data with PhyloToL to remove contaminating sequences prevalent in analyses of single cells that have been isolated from complex environments (e.g., the benthos). Determining foraminiferal species can be challenging, particularly given the presence of cryptic species in some groups and the lack of images for some single-cell data from GenBank. For the analyses here, we refer to each cell based on its morphological identification at the time of isolation plus inferences from analyses of assembled rDNA contigs that were captured as bycatch in these poly-A selected transcriptomes (see methods; File S1 available through Figshare at https://doi.org/10.6084/m9.figshare.28083020). Intriguingly, one cell that was identified morphologically as belonging to the genus *Trochammina* (Sr_rh_Ti04, File S1 available through Figshare at https://doi.org/10.6084/m9.figshare.28083020) falls on a long branch among the early-diverging monothalamids in our phylogeny (see below) and is represented by an 18S-rDNA sequence that is most similar to the monothalamid genus *Vellaria*. We infer that the *Trochammina* test (shell) that we isolated was inhabited by a monothalamid that was “squatting” within the otherwise empty test, a phenomenon previously reported for other monothalamids (49, 50), and we refer to this cell as “*Vellaria*-like.” Combining our data with those on Genbank, 10 cells fall into eight genera among the non-monophyletic single-chambered monothalamids, including one cell found in a freshwater habitat (taxon code Sr_rh_FF02; File S1). Twenty-six cells are from 12 genera of Globothalamea, organisms that build tests out of complex, “globular” chambers, while 10 cells represent 5 genera of Tubothalamea, cells that build tests in a roughly tubular shape. In addition, we include a single species from each of three genera of the deep-sea Xenophyophorea, previously deposited on GenBank (Table 1; File S1 available through Figshare at https://doi.org/10.6084/m9.figshare.28083020).

Given that each foraminifera was hand-picked from the environment and came with an associated microbiome (e.g., ectobionts, symbionts), we put considerable effort into identifying host sequences and removing “contaminants” (e.g., the microbiome). Curation of protein-coding sequences relied on a phylogenomic approach based on multiple sequence alignments and gene trees built by PhyloToL (45) and used methods developed for our study of interdomain lateral gene transfer (51). These methods included evaluating compositional bias and codon usage and inspecting both alignments and sister relationships in phylogenies (i.e., to identify fungal or diatom sequences [see Materials and Methods; File S4 available through Figshare at https://doi.org/10.6084/m9.figshare.28179515]). This enabled us to remove both poor-quality and contaminating sequences, with the latter including cross-contamination between cells sequenced on the same plate (i.e., index hopping [52, 53]) and sequences from the microbiomes of the foraminifera (e.g., references 54 and 55). After several rounds of curation, we retained 41,291 sequences that represent 1,044 gene families (Files S1 and S2 available through Figshare at https://doi.org/10.6084/m9.figshare.28083020 and https://doi.org/10.6084/m9.figshare.28083083, respectively).

### Phylogenomic analysis

To infer the taxonomic distribution of compositional bias and codon/amino acid preferences, we constructed a phylogeny for our 49 taxa. Importantly, this species tree encompasses what is likely more than 600 million years of evolution (40, 41), and therefore represents a concomitant amount of biodiversity that is unusual for molecular evolution studies of microeukaryotes. Placing compositional bias in the context of phylogeny can reveal forces driving bias in eukaryotes, as has been well documented in *Plasmodium* (56), and more recently for multiple lineages of phytoplankton (39).

**Fig 2:**
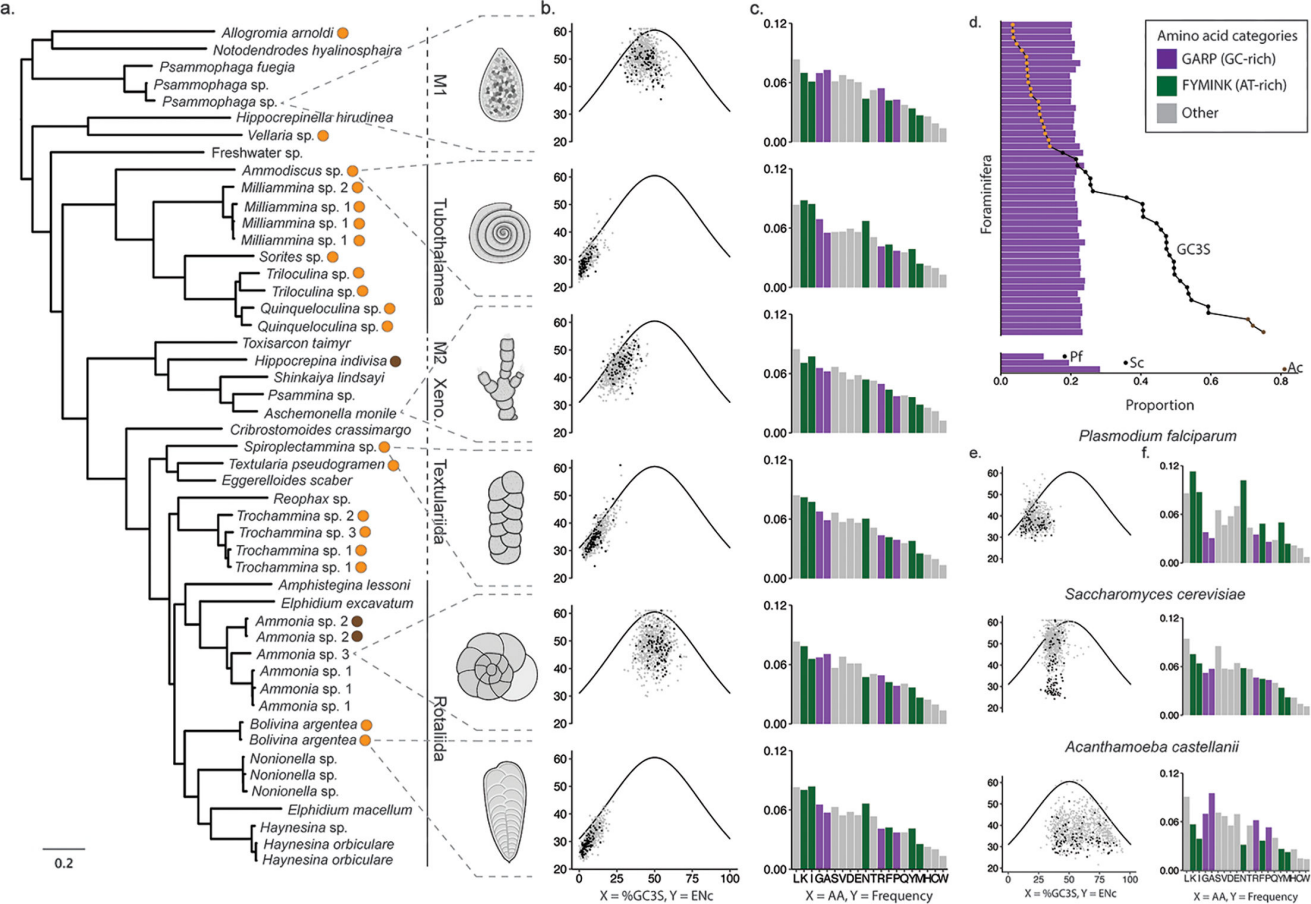
Amino acid usage is highly conserved across foraminifera, despite extreme non-monophyletic compositional bias in some taxa. (a) A maximum likelihood tree constructed from a concatenated alignment of 171 gene families conserved among foraminifera. Estimating phylogenetic relationships among the 49 foraminifera used in this study demonstrated that highly AT-rich taxa (with a median silent site GC content of less than 15%, marked by an orange circle) fall in several non-monophyletic lineages. Brown circles correspond to species with GC content above 70%. Low GC content is reflected in points lying to the far left on the GC3S vs. ENc plots. The clade Globothalamea comprises the Textulariida and the Rotaliida. Branch length (scale bar in bottom left) measures amino acid substitutions per site. (b) The ENc tends to fall below the null expectation. Black points indicate genes in the top 10% of expression, all other genes are shown in gray. (c) Despite the variation in GC content, amino acid usage is conserved in foraminifera, in contrast to the trends in the non-foraminifera taxa that were selected for variation in composition bias (e). In these non-foraminifera species, amino acid usage responds to GC pressure, with, for example, FYMINK (purple) amino acids overused in the AT-rich *Plasmodium falciparum* and GARP (green) amino acids overused in GC-rich *Acanthamoeba castellanii* (d, e).

We generated a species tree by concatenating 171 genes chosen based on their shared presence across our focal 49 cells. The resulting phylogeny (Fig. 2a) is consistent with recent reconstructions, including a study based on 199 genes from 68 species (42). We recover monophyletic Globothalamea, Tubothalamea, and Xenophyophorea nested among single-chambered polyphyletic “monothalamid” lineages (Fig. 2a). We find robust support for the sister status of Miliolida and Spirulillida (the former absent from (42)) within the Tubothalamea; we also find that lineages within Textulariida are paraphyletic with respect to other Globothalamea, which is consistent with prior rDNA analyses (41). As with other molecular analyses (41, 42), the greatest genetic diversity within foraminifera falls among monothalamids that are found throughout the inferred phylogeny (Fig. 2a).

### Patterns at the nucleotide level: compositional bias and codon usage

To evaluate compositional bias within transcripts, we calculated the GC content at fourfold degenerate silent sites (GC4) and found that *per* species median values among foraminifera range from 2.3% to 76% (File S1 available through Figshare at https://doi.org/10.6084/m9.figshare.28083020). Of the 28 genera included in the study, we categorized 11 as exhibiting extreme AT bias (hereby referred to as AT-biased taxa), with a median GC4 less than 15% (2.3%–13% GC; yellow dots, Fig. 2a). We categorize nine genera as “intermediate” (17%–39% GC4), nine as “non-biased” (40%–60% GC4), and three as GC-biased (70%–76% GC4, brown dots, Fig. 2a). Intriguingly, the AT-biased genera fall among multiple non-monophyletic lineages and include early-diverging monothalamids (*Allogromia* and *Vellaria*-like), Tubothalamea (*Ammodiscus, Miliammina, Quinqueloculina, Sorites,* and *Triloculina*), textularids (*Spiroplectammina, Textularia,* and *Trochammina*), and the Globothalamea genus *Bolivina* (Fig. 2a). We also see substantial variation within some putative genera; for example, *Ammonia* sp. 1 and 3 have a GC4 content of 46–49% while *Ammonia* sp. 2 is among the most GC-rich taxa (71%–72% GC4). We note that though GC4 captures only fourfold and sixfold amino acid families, in the AT-biased taxa, GC content at the third positions of all codons excluding those encoding tryptophan and methionine (GC3S) corresponds very closely to GC4; in taxa with higher GC content, third-position twofold and threefold degenerate sites tend to be more AT-biased than third-position fourfold sites (Fig. S16).

We assessed the relationship between nucleotide composition and codon usage and related these patterns to expression level. Using the equations of Wright (57), we find that the effective number of codons (ENc) corresponds well with the degree of compositional bias as measured by the GC3S, as more highly biased taxa use fewer codons (Fig. S2; File S1 available through Figshare at https://doi.org/10.6084/m9.figshare.28083020). To exemplify patterns among foraminifera, we plot data for six exemplar lineages across our inferred phylogeny of foraminifera (Fig. 2a and b), providing data for all cells in Fig. S2. The taxon with the most extreme bias, *Ammodiscus* sp., has a median GC3S of 2.6% and a median ENc of 27.92 codons; this is lower than the null expectation for a taxon with 0% GC3S as there are 31 codons with only A or T at a silent third position (solid line, Fig. 2b). As calculated based on Wright (57), this null expectation assumes that codon usage is driven only by silent-site GC content (which does not capture all possible neutral drivers of codon usage). Species with higher GC content like *Ammonia* sp. 2 also use fewer than expected codons (Fig. S2). In fact, in all of the foraminifera we examined, we observe a distribution of ENc values lower than the null expectation (Fig. S14a), consistent with selection at the codon level (see below), though also potentially with other dynamics such as context-dependent mutation (58, 59).

**Fig 3:**
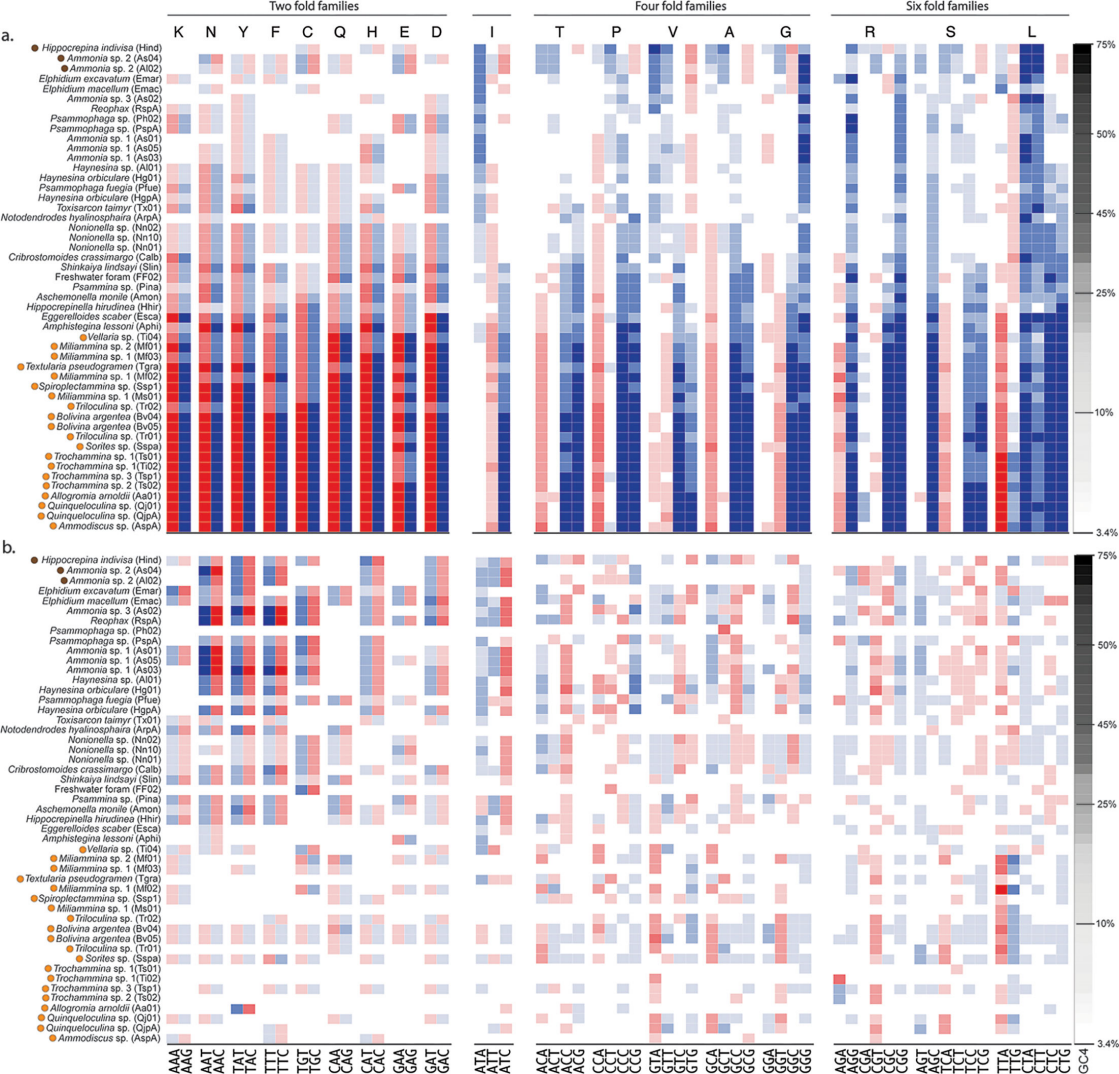
Usage of synonymous codons and the correlation of codon usage with expression vary systematically among taxa. (a) Shows observed codon usage frequency data, calculated as the number of times a codon is used divided by the total number of times the corresponding amino acid is used in that taxon. White indicates no bias (50% in a twofold family, 25% in a fourfold family, etc.), red indicates high frequency, and blue indicates low frequency. Among the striking patterns here are the near-zero values for many codons in the most biased taxa (bottom of panel, File S13 available through Figshare at https://doi.org/10.6084/m9.figshare.28083002). Here we see patterns consistent with selection for codon usage among all foraminifera; for example, for proline, the codon CCA is preferred over CCT while for isoleucine ATT is preferred over ATA. Amino acids are arranged in order of frequency, decreasing from left to right, and taxa are arranged in order of increasing AT bias (top to bottom; GC4 shown in grayscale bar on the right). (b) Shows the slope of codon frequency with log-scaled TPM, putatively representing the degree of selection for codon usage. Red indicates a positive slope (the codon is more frequent in highly expressed genes), and blue indicates a negative slope (File S13 available through Figshare at https://doi.org/10.6084/m9.figshare.28083002).

Extreme patterns in codon usage are also evident in analyses of codon usage frequencies for each amino acid, and we observe near-zero values in the most biased of the foraminifera we investigated (Fig. 3a), which are unlikely under explanations that rely on neutral processes alone. For example, across all genes included in this study, the extremely AT-biased monothalamid *Allogromia arnoldi* (GC4 median = 4.0%) uses the GC-biased codons, CGG and CGC, at three and eight of the 1,845 arginine sites, respectively (0.16% and 0.43%), whereas the most AT-biased arginine codon, AGA, is used at 1,213 sites (65.75%; Fig. 3a; File S1 available through Figshare at https://doi.org/10.6084/m9.figshare.28083020).

### Substitution bias is insufficient to explain compositional bias in foraminifera

**Fig 4:**
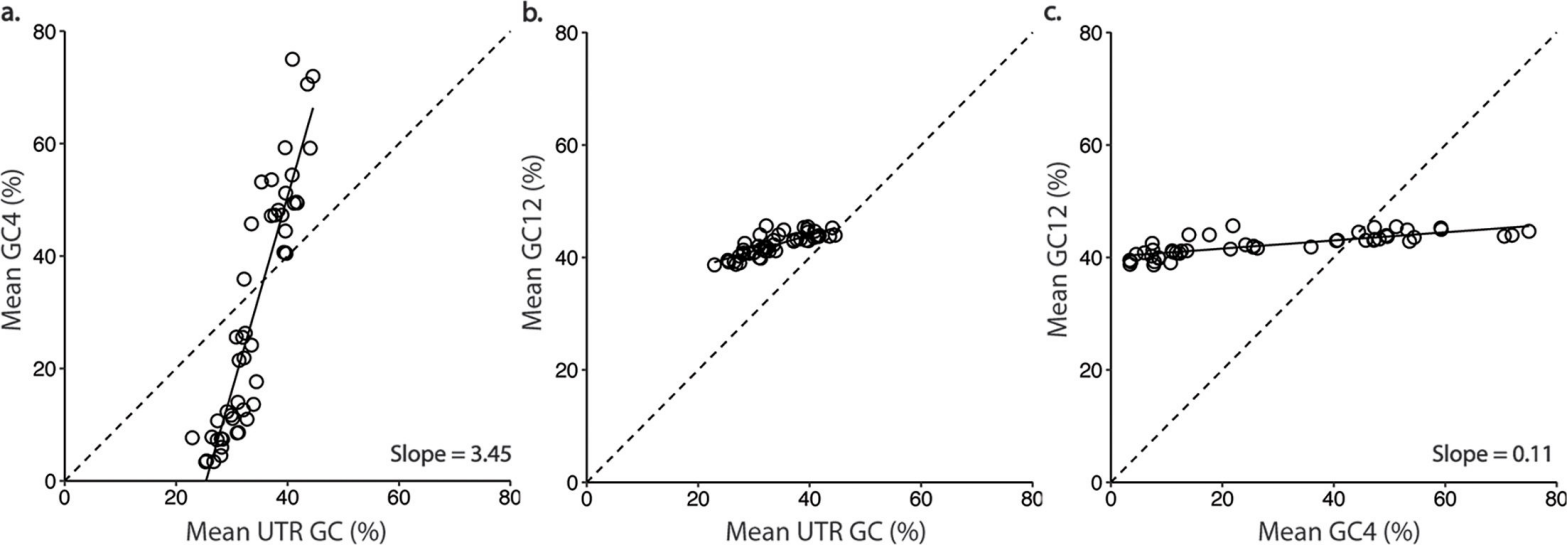
Neither substitution bias nor constraint is sufficient to explain the patterns in compositional bias in foraminifera. (a) While the GC content of UTRs does correlate positively with that of silent sites in ORFs, the range in the UTRs is much more restricted, indicating that there may be other drivers of compositional bias than biased substitution dynamics. (b) GC12 correlates with UTR GC content better than GC4 does, and is always greater than UTR GC content, except when the optimal GC12 (~45%) as determined by selection for amino acid usage matches the GC content of the UTRs. (c) Mean GC12 correlates with GC4 with a very shallow positive slope, indicating that GC12 is under much greater constraint than GC4 and/or is not subject to the same forces driving extreme composition. All corresponding taxon-level statistics can be found in File S1, available through Figshare at https://doi.org/10.6084/m9.figshare.28083020.

To estimate the degree to which substitution bias drives the composition of foraminiferal transcripts, we compare the nucleotide composition of coding domains to that of their associated UTRs (both 5′ and 3′), as UTRs are expected to more closely reflect mutational processes due to lower functional constraint (though see below). We find that the average GC content of foraminiferal UTRs lies between ~25%–45%, a range much smaller than that of the GC content at silent sites for the same taxa, which varies from 4% to 68% GC (File S1 available through Figshare at https://doi.org/10.6084/m9.figshare.28083020, Fig. 4a). We evaluated the relationship between compositional bias of GC4 and UTRs for each foraminifera (Fig. S14) and estimated the slope of this relationship across all foraminifera as 3.5 (Fig. 4a). The positive slope indicates that mutational processes may explain some of the extreme composition at silent sites in our AT-biased taxa. Next, we assessed the relationship between GC content in the non-synonymous 1st and 2nd codon positions (GC12) and GC content in UTRs across foraminifera, with the expectation that the range of GC12 would be narrower given functional constraints on proteins. Across all foraminifera, GC12 varies only from 38.7% to 45.7% and the slope of the relationship between GC12 and UTR GC content is 0.28 (Fig. 4b). Finally, we calculate the relationship between GC12 of GC4 and find a shallower slope of 0.11, consistent with functional constraint operating on GC12 content (Fig. 4c).

It is unlikely that UTRs are truly selectively neutral as (i) they contain regulatory elements (60) and (ii) we find evidence that UTRs are constrained. For example, both 5′ and 3′ UTRs show strong GC skew, and the 3′ UTRs prefer T and G whereas the 5′ UTRs prefer A and C (Fig. S9). Furthermore, single nucleotide frequencies in UTRs correlate with expression level as measured by transcripts per million (TPM): in 5′ UTRs, the usage of C and A correlates positively across all taxa, and that of T and G correlates negatively, whereas in the 3′ UTRs the correlation is generally the reverse but varies systematically with the average GC content of the taxon (Fig. S9).

To further account for neutral drivers of codon usage bias, we investigated patterns of dinucleotide usage, as context-dependent substitution bias could influence codon usage in a manner not detectable in an analysis with a null expectation based on single-nucleotide dynamics alone (61, 62). To this end, we adapted a recently developed metric of dinucleotide bias (63) that controls for single-nucleotide and amino-acid frequencies, as well as biases specific to codon positions, which we call site-specific synonymous dinucleotide usage (SSDU; see methods). Using this metric, we find that many dinucleotides show systematic patterns of under- or over-usage relative to the null expectation (Fig. S15d). Across all taxa, SSDU (in ORFs) correlates well with dinucleotide bias (controlling for single nucleotide frequency) in UTRs (slope = 0.68; Fig. S15c), consistent with a hypothesis of neutral context-dependent substitution biases (though see caveats relating to treating UTRs as neutral above). However, the direction of bias for some dinucleotides (such as AA and GA) goes in opposite directions in the ORFs and the UTRs across almost all taxa, indicating other dynamics (Fig. S15e). In some cases, such as for CT and GA at codon position 2, the direction of dinucleotide preference differs between AT-biased and other taxa; however, for the majority of dinucleotides, bias is directionally the same across all taxa (Fig. S15e), making it unlikely that it is driving differences in codon usage between species.

### Trade-offs between substitution bias and constraint as assessed by neutrality plots

**Fig 5:**
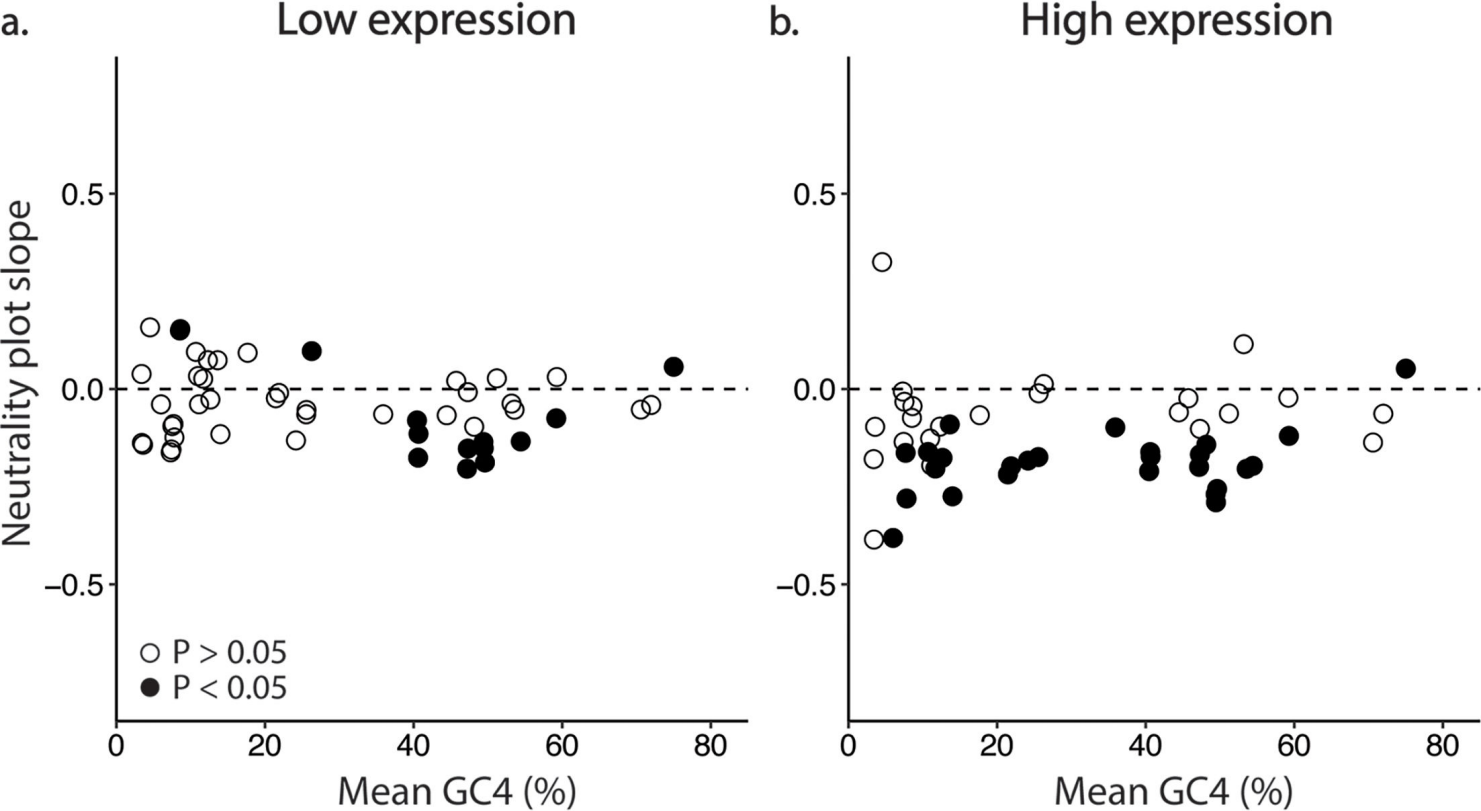
Neutrality plots show negative slopes, which are most extreme in highly expressed genes. Under substitution bias only, the expectation is a slope of 1, though this is usually mitigated by constraints on amino acid usage, yielding a positive slope of less than 1; negative slopes are unusual. (a) In genes falling in the bottom decile of expression (calculated per taxon), neutrality plots (GC12 vs. GC4) show, in foraminifera, slopes of about zero or less than 0, and in some cases significantly less (black dots). (b) In genes that fall in the top decile of expression, even more, foraminifera show significantly negative slopes. Corresponding per-taxon graphs can be found in Fig. S5.

To assess the dynamics between substitution bias (i.e., drivers of nucleotide composition) and functional constraints on proteins, we evaluated the correlation of GC12 with GC4 for each transcript. We refer to these plots as “neutrality plots” (64) for the sake of consistency with existing literature and without assuming the neutral nature of mutations (65). Neutrality plots describe the relationship between GC12 and GC4, with an expected slope of 1 if substitution biases alone drive GC bias, while functional constraints on proteins would drive this slope toward 0 (64, 66). Surprisingly, the slopes of neutrality plots across all foraminifera tend to be near-zero or significantly negative (Fig. 5; Fig. S5). Interpreting these data is challenging as a negative slope signifies complex dynamics, with more GC-rich non-silent sites (GC12) associated with more AT-rich silent sites (GC4). Also surprising is that this pattern persists across all foraminifera, regardless of bias in GC4.

To test the degree to which selection plays a role in shaping patterns here, we generated neutrality plots for genes in the bottom (Fig. 5a; Fig. S5) and top (Fig. 5b) decile of expression; by the mutation-selection-drift theory, those in the latter category are likely under stronger selection (26, 67, 68). In lowly expressed genes, we see a mix of negative and positive slopes, with most not significantly different from zero. By contrast, we observe negative slopes in highly expressed genes for all but four foraminifera, with significantly negative slopes in about half of the cells, and a significantly positive slope in only a single cell (the most GC-biased taxon; Fig. 5b). In the AT-biased foraminifera, negative neutrality plot slopes for AT-biased foraminifera are consistent with an intersection of two phenomena: positive selection for optimal codons (in general, AT-biased at silent sites) and positive selection for a specific amino acid distribution (see below). In taxa with higher GC4, the driver of negative slopes (Fig. 5) across all expression levels is unclear.

A negative correlation between GC4 and GC12 is rarely reported in eukaryotes, and in these cases, often only the magnitude of the slope is considered, with deviance from a slope of zero attributed to substitution bias (69–72). The most common trend in eukaryotes is a positive correlation between GC12 and GC4 as expected for a trade-off between substitution bias and functional constraints, and examples here abound (73–77). A study on the eukaryotic parasite *Leishmania* (66) argues that negative neutrality plots in this taxon are due to discrepancies in transition *versus* transversion dynamics between twofold and fourfold families as the negative correlation becomes much weaker when only fourfold families are considered. In contrast, we do not observe a substantial difference in correlation between synonymous site GC content and GC12 when considering twofold vs. fourfold families (Fig. S17). In surveying other lineages, the *Leishmania* study found frequent negative correlations on neutrality plots for prokaryotes, while a similar pattern is seen only in two more of the eukaryotes surveyed (*Schistosoma japonicum* and *Neurospora crassa*) (66).

It remains unclear what drives the negative correlation between GC4 and GC12 as seen in neutrality plots for foraminifera, and particularly for GC-neutral foraminifera. It is possible that there are hidden neutral factors at play here, as suggested by *Leishmania* (66). For example, if the ancestor of foraminifera was AT rich, then it could be that the relatively GC-neutral species are in a transitional state and therefore not in mutational equilibrium (64). Though this could, in theory, yield a negative correlation between GC12 and GC4 if genes evolved at differing rates, a full explanation of these patterns awaits additional experimental and theoretical work.

### Selection of optimal codons

**Fig 6:**
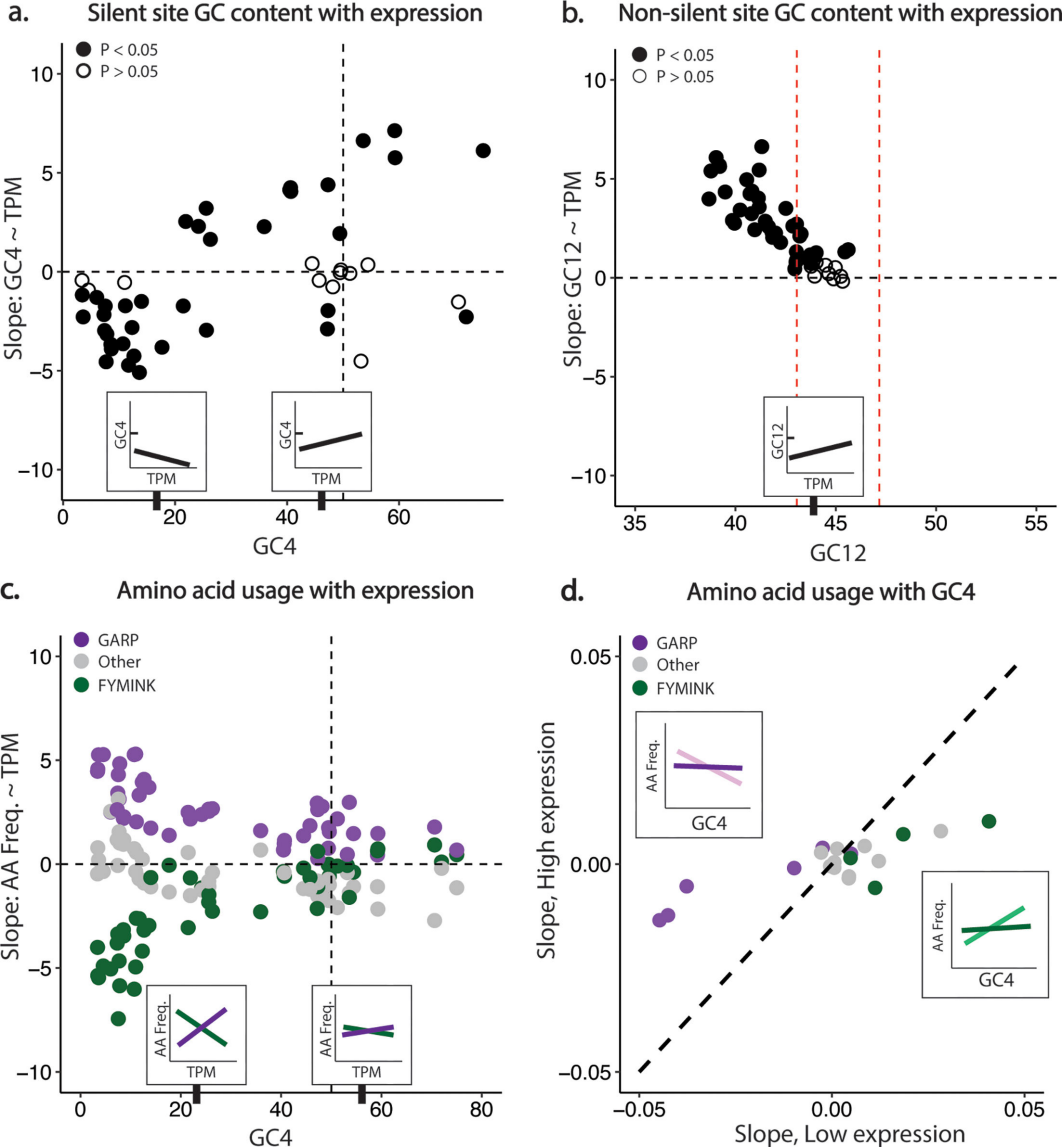
Composition and amino acid usage correlate with expression level. (a) Though highly expressed genes are more AT rich at silent sites in the most AT-rich taxa, the inverse is true for species with a mean GC4 of ≥25% (see Fig. S6 for details). (b) The GC content of non-silent sites correlates positively with expression in nearly all of the foraminifera, with the magnitude of the slope greater in taxa with lower GC12 content; this indicates that lowly expressed genes are further from the optimal GC12 content (see Fig. S6 for details). Red dashed lines are the lower and upper bounds across all taxa of the hypothetical GC12 calculated by assuming equal usage of codons within each amino acid (i.e., no codon usage bias), and then calculating the resulting GC12 using the true amino acid frequencies. (c) Patterns of amino acid usage of highly expressed genes vary with GC4 in biased taxa, as shown by the negative correlation of AT-rich amino acid frequency (FYMINK, green points) and accompanying positive correlation of GC-rich amino acid frequency (GARP, purple points). This trend becomes less pronounced in unbiased and GC-rich taxa. (d) While in lowly expressed genes (bottom two deciles of TPM), amino acid usage shows the effect of GC pressure, the correlation between amino acid usage and silent-site GC content is much lower in highly expressed genes (top two deciles of TPM). This indicates strong pressure to conserve a specific amino acid usage distribution. Each point is an amino acid, and the axes measure the slope of the mean amino acid frequency with mean GC4 across all foraminifera. Inserts in each graph exemplify per taxon patterns, with the light and dark lines in (d) indicating lowly and highly expressed genes, respectively.

Another well-documented phenomenon that drives composition within coding domains is the selection for a lineage-specific optimal set of codons, which is most evident in highly expressed genes as explained by the mutation-selection-drift model (78–80). It can be difficult to disentangle selection at the codon level from nucleotide, dinucleotide, or even amino-acid level pressures (81). Here we focus on both the correlation of codon usage with expression level as measured by TPM and discrepancies in codon usage from the expectation given silent-site and UTR GC content, an approach justified by the lack of data on tRNA abundance for any foraminifera. As a first step, we assess the relationship between GC4 and expression and find that in both intermediate and non-biased foraminifera there is a significant positive correlation between expression and GC4 of transcripts (Fig. S6; Fig. 6a). In AT-biased foraminifera, this trend is reversed, as GC4 correlates negatively with expression, consistent with positive selection for an optimal set of (on average) AT-rich codons in these lineages (Fig. S6; Fig. 6a).

Patterns of nucleotide substitution are insufficient to explain this relationship between GC4 and expression. We calculated the correlation between codon usage frequency and TPM as a proxy for selection on codon usage under the mutation-selection-drift model and found significant relationships widespread among foraminifera (Fig. 3b). Consistent with the analysis of GC4 and TPM, we observe that in AT-biased taxa, AT-ending codons tend to correlate positively with TPM and GC-ending codons correlate negatively, whereas the trend is reversed in GC-neutral and GC-biased taxa. We also see a preference for A-ending codons (over T-ending codons) in some amino acids and the reverse in other amino acids. For example, GTA and GCA tend to correlate significantly positively with TPM in AT-biased taxa, while the corresponding GTT and GCT correlate significantly negatively, whereas the opposite is true for GGT and GGA. Notably, these trends are not wholly explicable by dinucleotide preferences: for example, the TA dinucleotide is in general underused and TT is overused in AT-biased taxa as measured by SSDU, inconsistent with GTA being the preferred codon over GTT as suggested by the analysis of the correlation of codon frequency with TPM (Fig. S15a).

Similarly, as measured by relative synonymous codon usage (RSCU), AT-biased foraminifera exhibit inconsistent preferences between A and T at silent third positions. For example, in isoleucine, ATT is generally preferred over ATA, whereas for threonine ACA is preferred over ACT (Fig. 3). In all foraminifera, there is on average positive AT skew and negative GC skew at silent sites in coding regions; in AT-rich taxa, this skew is far greater in magnitude at ORF silent sites than it is in UTRs (Fig. S10). We calculated the expected distribution of codon usage given the usage of individual nucleotides and observed that intermediate and non-biased taxa show codon usage inconsistent with predictions based on both overall silent-site and UTR nucleotide content (Fig. S12), though neutral context-specific biases that are not captured by null expectations at the single-nucleotide level could also be playing a role here. We find that in AT-biased taxa, codon usage tends to match silent-site nucleotide content fairly closely in the twofold amino acid families, while fourfold families show a more complex pattern (Fig. S12a). In taxa with higher GC content, codon usage in twofold families diverges from the expectation given silent site GC content, as a more AT-rich codon set is preferred (Fig. S12a). Importantly, neither differences in raw codon frequencies nor the correlation of codon usage with expression correspond to accepted taxonomic boundaries. We find wide variations in codon usage and putative selection within clades (e.g., Rotaliida), and some non-monophyletic groups separated by hundreds of millions of years of evolution show very similar patterns in both their (extreme) codon usage frequencies (Fig. S14a) and the correlation of codon usage with expression (Fig. S14b).

We also find that in AT-biased taxa, codons closer to the 5′ and 3′ ends of a gene have a higher average silent-site GC content than those further from CDS-UTR boundaries (Fig. S11). This is consistent with the use of “non-optimal” codons to influence the physical process of translation, perhaps by increasing accuracy (20, 82, 83). This pattern could also be evidence of a strategy to mitigate the interference of mRNA secondary structure with ribosome binding, reduce the rate of translation abortion and downstream ribosomal traffic (20, 84–87), or aid in co-translational folding (88). Strategies such as the above may be especially important in taxa with strong constraints on amino acid usage, as appears to be the case in foraminifera. Lastly, we observe that the codon TAG is underused in all taxa, whereas the use of TAA and TGA varies with composition (Fig. S13). A similar observation has been made in other situations, including across prokaryotic genomes and in human isochores, and has been dubbed the “TAG paradox” (89).

### Selection for amino acid usage

Amino acid usage often corresponds to underlying nucleotide composition as a result of a phenomenon sometimes termed “GC pressure” (17, 18, 90). In AT-biased species, this is expected to lead to an overabundance of amino acids with AT-rich codons (phenylalanine (F), tyrosine (Y), methionine (M), isoleucine (I), asparagine (N), and lysine (K); abbreviated as FYMINK) while GC-biased taxa see an increased presence of glycine (G), alanine (A), arginine (R), proline (P); abbreviated as GARP. Consistent with this, the AT-rich *P. falciparum* (91, 92) and *D. discoideum* (93) show a strong preference for FYMINK amino acids, and, inversely, the GC-rich *A. castellanii* is GARP-biased (Fig. 2E).

To investigate the forces behind the conserved amino acid usage in foraminifera (Fig. 2C), we analyzed relative amino acid usage (frequency of “FYMINK” vs “GARP” residues) in the context of expression, with the expectation that more highly expressed genes would reflect lineage-specific “preferences” (i.e., are more likely to be driven by selection) as compared to lowly expressed genes. Indeed, genes in the bottom two deciles of expression show an effect of GC pressure, as in these genes the transcriptome-wide usage of GARP and FYMINK amino acids correlates positively and negatively with average taxon silent-site GC content, respectively (X-axis, Fig. 6d). By contrast, the correlation across taxa of FYMINK and GARP with average silent-site GC content in highly expressed genes is near zero for almost all amino acids (Y-axis, Fig. 6d). This implies that amino acid usage is largely independent of compositional bias, likely indicating that functional constraint is eliminating the effect of GC pressure in these genes, resulting in a highly similar distribution of amino acid frequencies across taxa with otherwise very divergent nucleotide and codon composition.

Consistent with selection driving amino acid usage, in AT-rich taxa, the frequency of FYMINK amino acids is reduced in more highly expressed genes, whereas GARP frequency is higher, with the remaining amino acids showing little or no correlation with expression level (Fig. 6c). Comparing across foraminifera, we find that selection for optimal amino acid usage drives GC12 to about 45%, with mutation driving AT-rich taxa away from this optimum. Taxa with lower GC12 (i.e., GC12 further from 45%) have a more positive correlation between GC12 and expression than taxa with GC12 closer to 45%, where the slope of GC12 and expression is generally not significantly different from zero (Fig. 6b). The GC content of UTRs, which we use as a proxy for estimating background substitution biases, with the caveat that UTRs exhibit evidence of constraint (Fig. S9), is less than GC12 in most foraminifera, with the exception of a few taxa where these measurements are about equal at 45% (Fig. 4c).

Across all foraminifera, proteins are enriched for the amino acids leucine, lysine, and isoleucine, while the amino acids methionine, histidine, cysteine, and tryptophan are rarest (Fig. 2C), a pattern also found in data from non-foraminifera plotted in Fig. 2E, and in diverse lineages across the tree of life (94–97). The factors behind the conserved pattern of amino acid usage across foraminifera (Fig. 2 and 6d) are unclear. One possibility is that amino acid usage may reflect synthesis cost; as originally estimated for *Escherichia coli* and *Bacillus subtilis,* the amino acids tryptophan (W), phenylalanine (F), and tyrosine (Y) have the greatest biosynthetic cost (96). One study extending these ideas to yeast demonstrated the high cost of these three amino acids plus methionine (M), histidine (H), and cytosine (C) (98). Interestingly, these amino acids (M, H, W, and C) are the least used among all the foraminifera in our study, suggesting that amino acid biosynthesis costs may be a factor driving patterns of amino acid usage and one that overcomes the GC pressure expected for our extremely biased species.

### Synthesis

Foraminifera present a conundrum, with highly conserved amino acid usage across multiple non-monophyletic clades with extreme compositional bias (GC4 as low as 2.3%; Fig. 2), interdigitated with GC-neutral lineages. The analyses presented here demonstrate that the forces driving nucleotide and amino acid compositions in foraminifera are complex and likely include substitution bias (Fig. 4) and context-dependent mutation (Fig. S15) as well as selection for both optimal codons and amino acids (Fig. 6b**;** Fig. S12). Despite these complex dynamics, foraminifera have conserved their amino acid usage across over ~600 million years of evolution (Fig. 2), suggesting some emergent property of the genome or proteome is a major driver of this macroevolutionary pattern.

Here we speculate on the possible drivers of these unusual patterns. One possibility is that the combination of extreme AT bias and observed amino acid usage found in multiple lineages of foraminifera represents an ancestral state and that the more GC-neutral lineages represent a change in mutation processes. Such a change in compositional bias is expected to lead to a concomitant increase in GC-rich proteins, as has been argued for *Plasmodium vivax* (56, 99); yet, we find highly conserved amino acids across all foraminifera (Fig. 2). Alternatively, foraminiferal lineages may have independently evolved extreme AT bias, perhaps as the result of selection for genome-scale variation as has been argued from *P. falciparum* (100) or in some way linked to the patterns of cyclical polyploidization that mark foraminiferal life cycles (40). Regardless, the constancy of amino acid usage is unexpected and suggests strong selection. It is possible that foraminifera have an extremely large effective population size (Ne), allowing weak per-site selection to maintain amino acid usage; yet, bacteria with large Ne still appear vulnerable to GC pressure (18).

Most broadly, the data presented here expand our knowledge of the evolution of codon usage and compositional bias, particularly with the discovery of a conserved amino acid distribution despite the highly AT-biased nature of codon usage in a clade of diverse microbial eukaryotes (Fig. 2D). We speculate that foraminifera are likely not alone in proving exceptions to the “rules” governing codon usage and compositional bias and that applying analyses like these to a larger set of eukaryotes will yield a more inclusive view of the dynamics of codon and amino acid usage, and may even point to factors that drove the evolution of the genetic code.

## MATERIALS AND METHODS

### Initial data curation

Single-cell transcriptome data used in this study had been previously processed using PhyloToL (45) and underwent additional curation steps as described in Cote-L’Heureux et al. (51). In sum, PhyloToL allows the identification of homologs for 13,630 gene families (GFs) as defined by OrthoMCL release 5.0 and chosen based on their presence in multiple eukaryotic lineages. PhyloToL translates sequences >200 bp, providing initial assignments to GFs based on similarity using USEARCH with an e-value cutoff of 1e-20. For this study, we initially identified the 1,200 gene families shared among the greatest number of single-cell transcriptomes from Foraminifera and found in at least one of the 28 cells sequenced in our lab that we had determined to be high quality in a pilot study. We excluded transcriptomes with few sequences assigned to gene families or that rarely appeared as sisters to closely related taxa in a control set of 35 conserved gene families. We further excluded some cells based on evidence of contamination, as represented by a wide range in their compositional bias and codon usage (e.g., Fig. S1). PhyloToL rigorously assessed the homology of sequences within each gene family using custom filters (45) and Guidance v2.02 (101) using a sequence score cutoff of 0.3.

To assess taxonomic names, we used BLASTn to compare all assembled transcripts to a curated database of foraminiferal rDNAs and the GenBank nr database. Here we use species names for cells whose inferred rRNA (≥500 bp sequence) is ≥99% identical to a reference and generic names for remaining cells whose inferred rRNA is ≥95% identical (File S1 available through Figshare at https://doi.org/10.6084/m9.figshare.28083020). Interpretation of the rRNA analysis should be done with caution as (i) the inferred rRNA sequences in this study were bycatch from poly-A selected transcriptomes; (ii) making strong taxonomic inferences solely on foraminiferan 18S rRNAs is challenging given the state of the data on GenBank, where some species are represented by only a portion of the 18S gene; and (iii) most described species of foraminifera still lack 18S-rDNA data. A list of the 49 cells included in the study with GenBank accession numbers and inferred names can be found in File S1, available through Figshare at https://doi.org/10.6084/m9.figshare.28083020.

### Gene-tree curation and sequence selection

After an initial round of curation of taxa, we selected 1,200 gene families for further analyses using 49 single-cell foraminiferal transcriptomes plus a set of outgroups to aid in identifying contaminating sequences (necessary as each foraminifera has an associated microbiome). We constructed individual gene trees using IQ-Tree (version 2.1.2 [102]) (“-m LG+G”) through the CIPRES Science Gateway REST API. We used custom Python scripts to select foraminiferan homologs for each gene tree by identifying foraminiferan sequences in monophyletic clades that contained samples from at least six foram species (with at least one representative from our lab) and that also contained no more than two non-foraminiferal sequences (often long branches). We then re-aligned the sequences from each selected sub-clade of foraminifera and reconstructed these highly reduced gene trees of foraminiferal sequences using IQ-Tree (-m LG+G). After inspecting trees by eye, we retained a total of 1,044 foraminifera-specific gene trees that serve as the backbone for subsequent analyses (Files S5 to S7 available through Figshare at https://doi.org/10.6084/m9.figshare.28082990, https://doi.org/10.6084/m9.figshare.28083065, and https://doi.org/10.6084/m9.figshare.28083053, respectively).

We performed a second round of data curation to address known issues with short-read Illumina data and *de novo* transcriptome assembly (e.g., cross-contamination by other samples, misassembly of transcripts, presence of partial transcripts). Following visual inspection of a subset of the alignments, cross-contaminant sequences were identified as nearly identical (≥99%) between taxa and for which there was a substantial difference in k-mer coverage depth, both indicators of bioinformatic bleed-through in short-read sequencing. These sequences were defined either with k-mer coverage <10 that also had a “paralog” (a sequence from the same cell) with coverage >20, or sequences with k-mer coverage <20 that had a “paralog” with k-mer coverage >100. Additional curation steps included removing (i) the lower k-mer coverage sequence from any pair of sequences from the same cell that was >95% identical or shared 50 base pairs of 100% identity; (ii) short transcripts defined as having <25% the length of the clade average; and (iii) paralogous sequences with a length <50% of the clade average. We also removed all but the representative with the highest k-mer coverage from clades of ingroup paralogs (i.e., sequences from the same cell that clustered on a tree) and sequences in which 30% of the sequence was inferred as gaps. Finally, we manually analyzed outliers from our GC3S and ENc distributions, removing all outlying points with k-mer coverage <10 or that had a non-outlying paralog with higher coverage. The efficacy of using k-mer coverage for sequence-level curation of single-cell transcriptomes, especially in the presence of pseudo-paralogs (multiple sequences from the same gene family within a taxon, but likely resulting from at least one or more of the sequences being contaminants), is exemplified in Fig. S1 and S2. Sequences surviving curation were re-aligned using Guidance.

To identify trends in substitution bias beyond coding regions, we analyzed the GC content of both 5′ and 3′ UTRs of transcripts following several curation steps that accounted for the incomplete nature of transcriptomic data: (i) only UTRs longer than 50 bp and shorter than 500 bp were considered; (ii) for a 5′ UTR to be included, the corresponding coding region must share a methionine with at least one other sequence in the alignment; and (iii) similarly, we only included 3′ UTRs that shared a stop codon in the same position as at least one other sequence.

### Phylogenetic inference

To provide an evolutionary framework for data interpretation, we estimated the evolutionary relationships among the 49 single-cell transcriptomes through a maximum likelihood analysis of a concatenated set of orthologs. From the 1,044 gene families used in the entire study, we concatenated 171 that had at least 30 of the 49 cells and no paralogy (i.e., that did not require us to identify orthologs), generating a matrix of 52,255 sites and 41,292 amino acids (File S12 available through Figshare at https://doi.org/10.6084/m9.figshare.28082978). The concatenated tree was built by partitioning the data so that each gene family matched the model chosen using ModelFinder (103) as implemented in IQ-Tree version 2 (102).

### Assessment of codon usage and compositional bias

Analysis of codon usage and compositional bias relied on custom Python scripts and published protocols. We calculated the effective number of codons following the equations provided in reference 57 and compared this to GC content at third position fourfold degenerate sites (i.e., GC4). We estimate expression using transcripts per million (TPM). We measured the number of reads per transcript with Salmon under default parameters plus the “--validateMappings” flag to enhance the sensitivity of transcript alignment (104) and then calculated TPM using the raw transcript length (rather than the effective length reported by Salmon) to reduce the distorting effect of short sequences. These same data were used to plot the relationship between coverage and varying base pairs (i.e., GC4 and each nucleotide separately) and to estimate neutrality plots (i.e., GC12 vs. GC4). All slopes were calculated using linear regression, and *P*-values were calculated using Spearman rank correlation with Benjamini-Hochberg correction where appropriate.

We present a new method to estimate site-specific dinucleotide usage (SSDU) by adapting the synonymous dinucleotide usage (SDU) metric proposed previously (63). The original SDU metric controls for single nucleotide usage and amino acid usage, but does not account for variation in single nucleotide usage by codon position. In our case, because we see such extreme variation in single-nucleotide usage between codon positions in many taxa, we adjusted the null expectation in the model to calculate expected codon usage using the frequency of single nucleotides at onefold, twofold, threefold, and fourfold sites. The program was implemented in Python and is available on Github (https://github.com/AudenCote/ForamCodonUsage).

## SUPPLEMENTAL MATERIAL

Supplemental figures mbio.03916-24-s0001.pdf Figures S1 to S17.

ASM does not own the copyrights to Supplemental Material that may be linked to, or accessed through, an article. The authors have granted ASM a non-exclusive, world-wide license to publish the Supplemental Material files. Please contact the corresponding author directly for reuse.
