# Supplementary material for: Lost in translation: conserved amino acid usage despite extreme codon bias in foraminifera
Source: mBio. 2025 Mar 5;16(4):e03916-24. doi: 10.1128/mbio.03916-24 (PMC11980380; doi:10.1128/mbio.03916-24)
Supplement: Supplemental figures — Figures S1 to S17. [file mbio.03916-24-s0001.pdf]

Supplemental figures for:

Lost in translation: conserved amino acid usage despite extreme codon bias in foraminifera

Auden Cote-L'Heureux[1], Elinor G Sterner[1], Xyrus X. Maurer-Alcalá[2], Laura A. Katz[1,3]*

S1 Fig. Coverage is a good predictor of cross-sample contamination, as exemplified by *Psammophaga fuegia* and *Hippocrepinella hirudinea*, which were sequenced as part of the same study**. (a)** Each dot represents a paralogous sequence in *P. fuegia* (Sr_rh_Pfue), which is known to be contaminated with RNA from *H. hirudinea* (Sr_rh_Hhir). Hollow points are sequences that are 95% identical to a sequence from the *H. hirudinea* transcriptome; filled points do hit an *H. hirudinea* sequence at >95% identity. Hollow points were removed from the study. **(b)** Each point represents a sequence from either *H. hirudinea* (solid points) or *P. fuegia* (hollow points) with a BLAST identity to a sequence from the other taxon >95%. The coverage gap between sequences is consistently large, indicating that one sequence (lowest covered) in each pair is likely a result of contamination from the other taxon.

S2 Fig. Silent-site GC content (GC4, X axis) and effective number of codons (ENc, Y axis) in all foraminifera. One plot for each taxon (codes corresponding to **File S1**), sorted by mean GC4 of each taxon. The number of transcripts (points) is given for each taxon in parentheses next to the taxon identifier. Black points are the top 10% expressed transcripts.

S3 Fig. Amino acid usage in all foraminifera. One plot for each taxon (codes corresponding to **File S1**), sorted by mean GC4 of each taxon. The number of transcripts (points) is given for each taxon in parentheses next to the taxon identifier. Amino acids are sorted by average

frequency. Green bars represent amino acids with AT-biased codons (FYMINK) and purple represent amino acids with GC-biased codons (GARP).

S4 Fig. UTR GC content generally correlates positively with silent-site GC content in ORFs. Plots are sorted by mean GC4 of each taxon. The total number of transcripts for each taxon is given in parentheses next to the taxon identifier. Only UTRs greater than 50bp and less than 500bp are considered. In some cases (e.g. Sr_rh_Ph02), there are very few robust UTR sequences available, leading to likely misleading correlations.

S5 Fig. Neutrality plots (GC12 vs. GC4) in all foraminifera. Plots are sorted by mean GC4 of each taxon; the number of transcripts (points) is given for each taxon in parentheses next to the taxon identifier. Transcripts in the top decile of expression for each taxon are in red, those in the bottom decile are in blue. The data are those underlying the slopes presented in **Fig 5a** (blue points) and **Fig 5b** (red points).

S6 Fig. Silent-site GC content (GC4) generally correlates negatively with expression (log-transformed TPM) in AT-biased taxa, and positively in more GC-neutral taxa. Plots are sorted by mean GC4 of each taxon; the number of transcripts (points) is given for each taxon in parentheses next to the taxon identifier. The slopes correspond to those presented in **Fig 6a**.

S7 Fig. Non-silent site GC content (GC12) generally correlates positively with expression (log-transformed TPM) in all foraminifera, though to a lesser extent in taxa with a mean GC12 close to 45%. Plots are sorted by mean GC4 of each taxon; the number of transcripts (points) is given for each taxon in parentheses next to the taxon identifier. The slopes correspond to those presented in **Fig 6b**.

S8 Fig. Relative effective number of codons (ENc) correlates complexly with expression. Each point represents a transcriptome; the Y axis measures the slope of relative ENc of each transcript *versus* the log-transformed TPM for the transcript. Relative ENc is defined as the observed ENc value of the transcript divided by the ‘null’ for the taxon minus 20, as calculated using that mean GC3S for each taxon (null calculated using formula given in Wright 1990). The mean GC3S for each transcriptome is given on the X axis. While AT-biased taxa generally show negative slopes, consistent with stronger selection for codon usage in highly expressed genes, some intermediate and non-biased taxa show positive slopes, potentially consistent with these taxa being in a transitional state.

S9 Fig. UTRs show interesting AT and GC skew. Only UTRs longer than 50bp and shorter than 500bp are considered. (A) Nucleotide frequencies for 5’ UTRs in each transcript transcript. (B) Nucleotide frequencies for 3’ UTRs in each transcript transcript. (C) Mean nucleotide frequencies for each taxon, relative to the mean GC4 of the taxon, for the 5’ and (D) 3’ UTRs. (E) The slope of nucleotide frequency *versus* expression (log-transformed TPM) for each taxon for the 5’ and (F) 3’ UTRs.

S10 Fig. AT and GC skew across CDS and UTRs. The Y axis measures the proportion of sites at the given distance from the start (left) and stop (right) codons that are the respective nucleotide. Each point represents a nucleotide position, and the lines are a sliding window average that capture the nearest 10% of all points. In AT-biased taxa, as exemplified by *Bolivina argentea* (top), there is strong positive AT skew which is lessened towards the 3’ boundary, and GC skew is weak and varies in direction between UTRs. In GC-neutral taxa, exemplified by *Ammonia* sp. (middle), there is positive AT and GC skew. This is also the case in GC-biased taxa, as exemplified by *Hippocrepina indivisa* (bottom), in which GC content converges towards 50% at the 5’ boundary and in which has AT-biased UTRs.

S11 Fig. Silent-site GC content (GC4) varies across CDS in some taxa. This is mostly clearly pronounced in AT-biased taxa (bottom), which have higher average GC content near start/stop codon boundaries than in the center of the CDS, potentially indicating usage of non-optimal codons in these regions. The Y axis measures the proportion of sites at the given distance from the start (left) and stop (right) codon that are silent (4-fold degenerate) and G or C.

S12 Fig. Codon usage varies from the null expectation given nucleotide usage patterns. Distance from the null (intensity of color) is calculated as the observed frequency (%) minus the null expectation (%). Only 2, 3, and 4-fold sites were considered in calculating null codon frequency from nucleotide usage. (A) Null expected codon frequencies were calculated using the nucleotide frequencies at third-position 4-fold degenerate sites in CDSs. The greatest differences lie in the more GC-neutral taxa, where in 2-fold families observed codon usage is more AT-rich than expected given silent site AT usage. (B) Null expected codon frequencies were calculated using nucleotide frequencies in UTRs; here the clearest differences are in the AT-rich taxa, with silent-site GC content very far from UTR GC content (see also **Fig 4a**).

S13 Fig. Stop codon usage varies by transcriptome composition. While the codon TAG (purple) is underused in all taxa regardless of compositional bias, the usage of TAA (green) and TGA (gray) is about equal in AT-biased taxa but the use of TGA increases and that of TAA decreases with mean GC4. The patterns shown by TAA and TGA are expected under substitutional biases, but the reasons behind the underuse of TAG are unclear.

S14 Fig. Codon usage and its correlation with expression are consistent across non-monophyletic clades. (A) Correspondence analysis on relative synonymous codon usage

(RSCU) across all codons and all taxa. (B) Correspondence analysis on the slope of RSCU with log-transformed TPM across all codons and all taxa. Points are colored by GC content at four-fold degenerate sites (GC4), and the shape of points corresponds to the main foraminiferal clades (see Fig 2a). (C) and (D) show the same data as in (A) and (B) respectively, but colored by clade to emphasize the interdigitation of non-monophyletic groups. In general, species do not organize by clade in either graph, and GC4 appears to explain the majority of the variance (horizontal axis in each subplot), consistent with the hypothesis that non-monophyletic groups of foraminifera have converged on similar highly biased sets of preferred codons.

S15 Fig. Dinucleotide usage is biased, but is not sufficient to explain codon usage. (A-B) The slope of RSCU with TPM (Y axis) for each codon (a proxy for selection), and the relative SDU (observed/expected) for the corresponding position-2 dinucleotide, exemplified for a GC-neutral (A) and AT-biased (B) taxon. Brown dots indicate GC-rich taxa and orange dots indicate AT-rich taxa, concordant with Fig. 2a. In particular, the direction of putative codon-level selection does not always correspond with the direction of the dinucleotide bias, as in the case of the AC-ending codons for Ammonia, and the TT-ending codons in Quinqueloculina. (C) Dinucleotide frequency in UTRs (relative to the expectation given single-nucleotide frequencies) correlates with SDU in ORFs, consistent with the hypothesis of neutral processes influencing dinucleotide frequency in ORFs. (D) Synonymous dinucleotide usage relative to the per-site null (see methods) at position-2 and bridge (across codons, position 3 to position 1) sites. Taxa (Y axis) are sorted in order of descending GC4, top to bottom. Yellow dots indicate AT-biased taxa, brown dots are GC-biased. (E) A heatmap showing whether the relative dinucleotide bias is directionally the same in UTRs and ORFs (observed/expected > 1 for both or observed/expected < 1 for both). Black indicates a match, gray indicates mismatch. Note in particular the column corresponding to dinucleotide AA, where ORF dinucleotide bias is consistently counter to that of UTRs, and the TA, TC, and TG columns, where the opposite trend

prevails nearly universally.

S16 Fig. Average GC3S plotted with GC4 across taxa, indicating that in general these values are very close (i.e., synonymous bias in 2-fold and 3-fold families is similar to that in 4-fold families).

S17 Fig. The slope of GC4 vs. TPM (X axis) as plotted with the slope of GC3S vs. TPM shows that neutrality plot slopes are generally similar in 2-fold and 3-fold vs. 4-fold families. In particular, the negative slope of neutrality plots does not appear to be primarily driven by bias in 2-fold families, as observed in *Leishmania* (66).

Figure S1

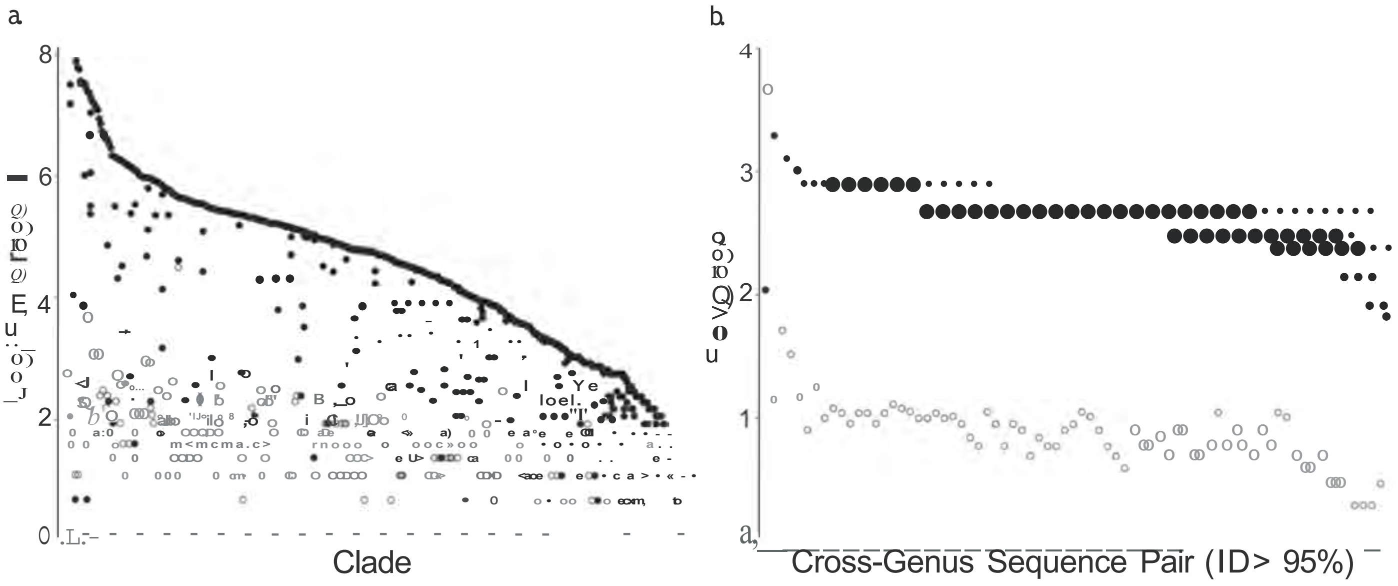

Fig. S2

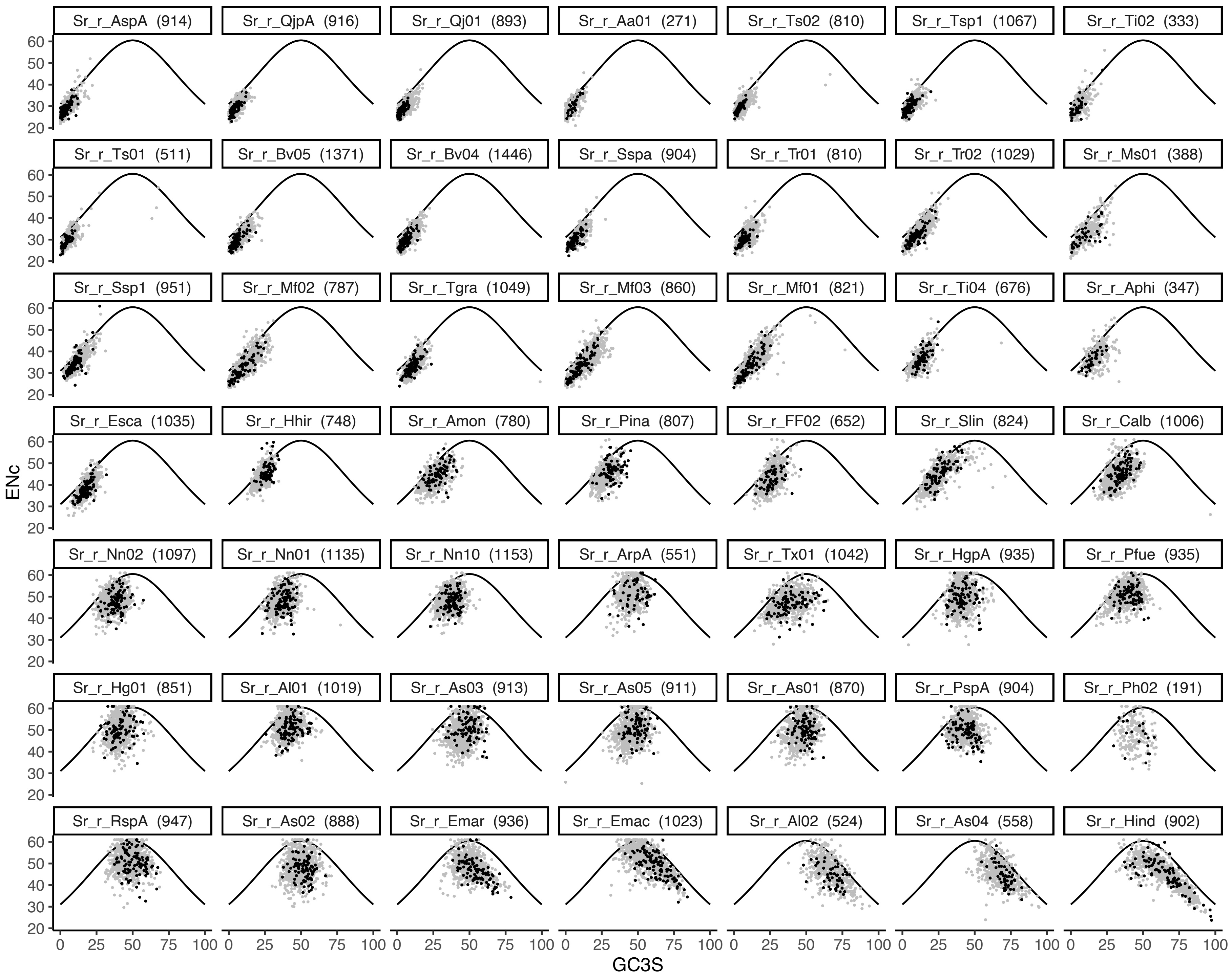

Sr_r_AspA (914)
Sr_r_QjpA (916)
Sr_r_Qj01 (893)
Sr_r_Aa01 (271)
Sr_r_Ts02 (810)
Sr_r_Tsp1 (1067)
Sr_r_Ti02 (333)
Sr_r_Ts01 (511)
Sr_r_Bv05 (1371)
Sr_r_Bv04 (1446)
Sr_r_Sspa (904)
Sr_r_Tr01 (810)
Sr_r_Tr02 (1029)
Sr_r_Ms01 (388)
Sr_r_Ssp1 (951)
Sr_r_Mf02 (787)
Sr_r_Tgra (1049)
Sr_r_Mf03 (860)
Sr_r_Mf01 (821)
Sr_r_Ti04 (676)
Sr_r_Aphi (347)
Sr_r_Esca (1035)
Sr_r_Hhir (748)
Sr_r_Amon (780)
Sr_r_Pina (807)
Sr_r_FF02 (652)
Sr_r_Slin (824)
Sr_r_Calb (1006)
Sr_r_Nn02 (1097)
Sr_r_Nn01 (1135)
Sr_r_Nn10 (1153)
Sr_r_ArpA (551)
Sr_r_Tx01 (1042)
Sr_r_HgpA (935)
Sr_r_Pfue (935)
Sr_r_Hg01 (851)
Sr_r_Al01 (1019)
Sr_r_As03 (913)
Sr_r_As05 (911)
Sr_r_As01 (870)
Sr_r_PspA (904)
Sr_r_Ph02 (191)
Sr_r_RspA (947)
Sr_r_As02 (888)
Sr_r_Emar (936)
Sr_r_Emac (1023)
Sr_r_Al02 (524)
Sr_r_As04 (558)
Sr_r_Hind (902)
ENc
GC3S

Fig. S3

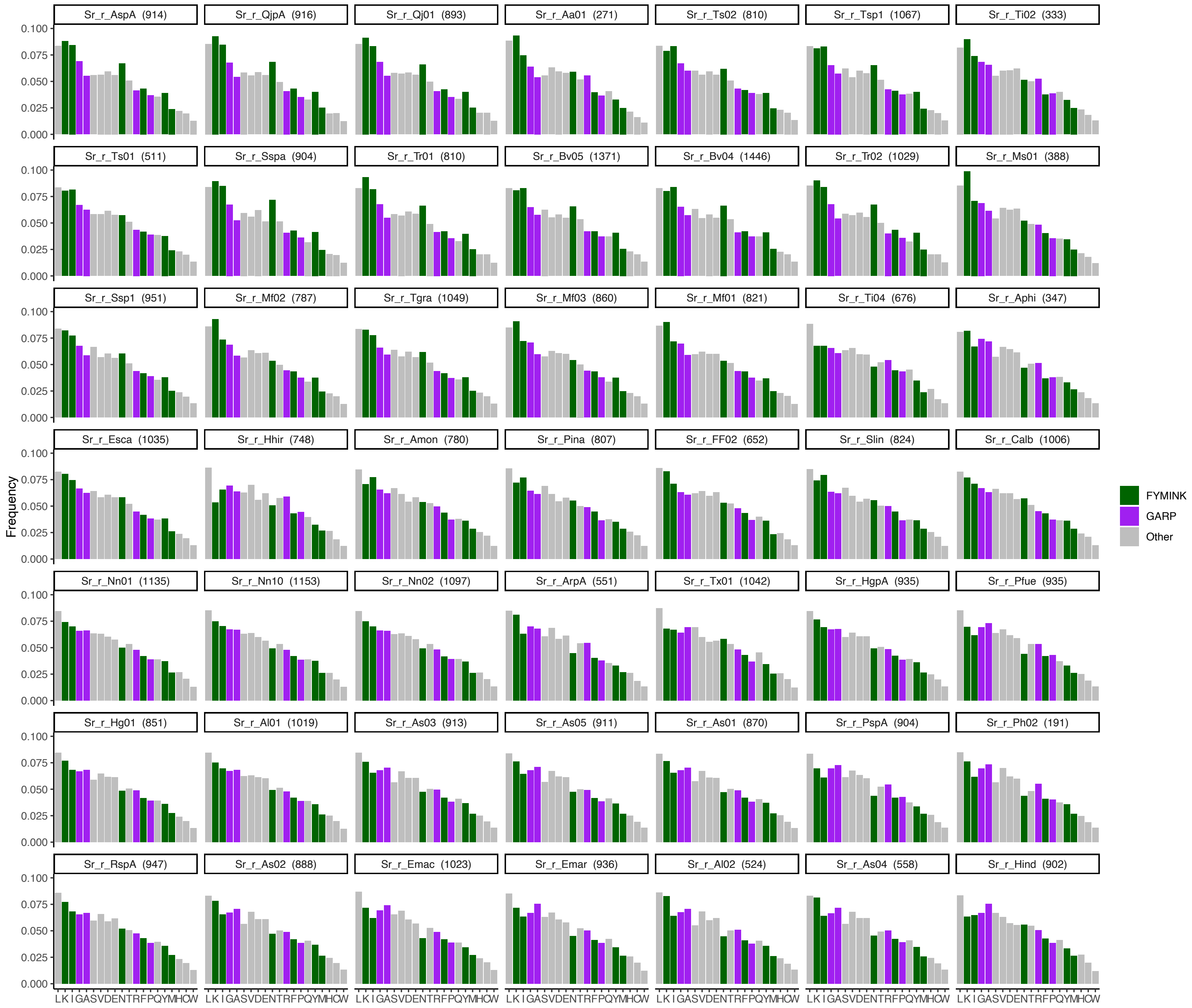
Sr_r_AspA (914)
Sr_r_QjpA (916)
Sr_r_Qj01 (893)
Sr_r_Aa01 (271)
Sr_r_Ts02 (810)
Sr_r_Tsp1 (1067)
Sr_r_Ti02 (333)
Sr_r_Ts01 (511)
Sr_r_Sspa (904)
Sr_r_Tr01 (810)
Sr_r_Bv05 (1371)
Sr_r_Bv04 (1446)
Sr_r_Tr02 (1029)
Sr_r_Ms01 (388)
Sr_r_Ssp1 (951)
Sr_r_Mf02 (787)
Sr_r_Tgra (1049)
Sr_r_Mf03 (860)
Sr_r_Mf01 (821)
Sr_r_Ti04 (676)
Sr_r_Aphi (347)
Sr_r_Esca (1035)
Sr_r_Hhir (748)
Sr_r_Amon (780)
Sr_r_Pina (807)
Sr_r_FF02 (652)
Sr_r_Slin (824)
Sr_r_Calb (1006)
Sr_r_Nn01 (1135)
Sr_r_Nn10 (1153)
Sr_r_Nn02 (1097)
Sr_r_ArpA (551)
Sr_r_Tx01 (1042)
Sr_r_HgpA (935)
Sr_r_Pfue (935)
Sr_r_Hg01 (851)
Sr_r_Al01 (1019)
Sr_r_As03 (913)
Sr_r_As05 (911)
Sr_r_As01 (870)
Sr_r_PspA (904)
Sr_r_Ph02 (191)
Sr_r_RspA (947)
Sr_r_As02 (888)
Sr_r_Emac (1023)
Sr_r_Emar (936)
Sr_r_Al02 (524)
Sr_r_As04 (558)
Sr_r_Hind (902)
Frequency
0.000
0.025
0.050
0.075
0.100
L K I G A S V D E N T R F P Q Y M H C W
FYMINK
GARP
Other

Fig. S4

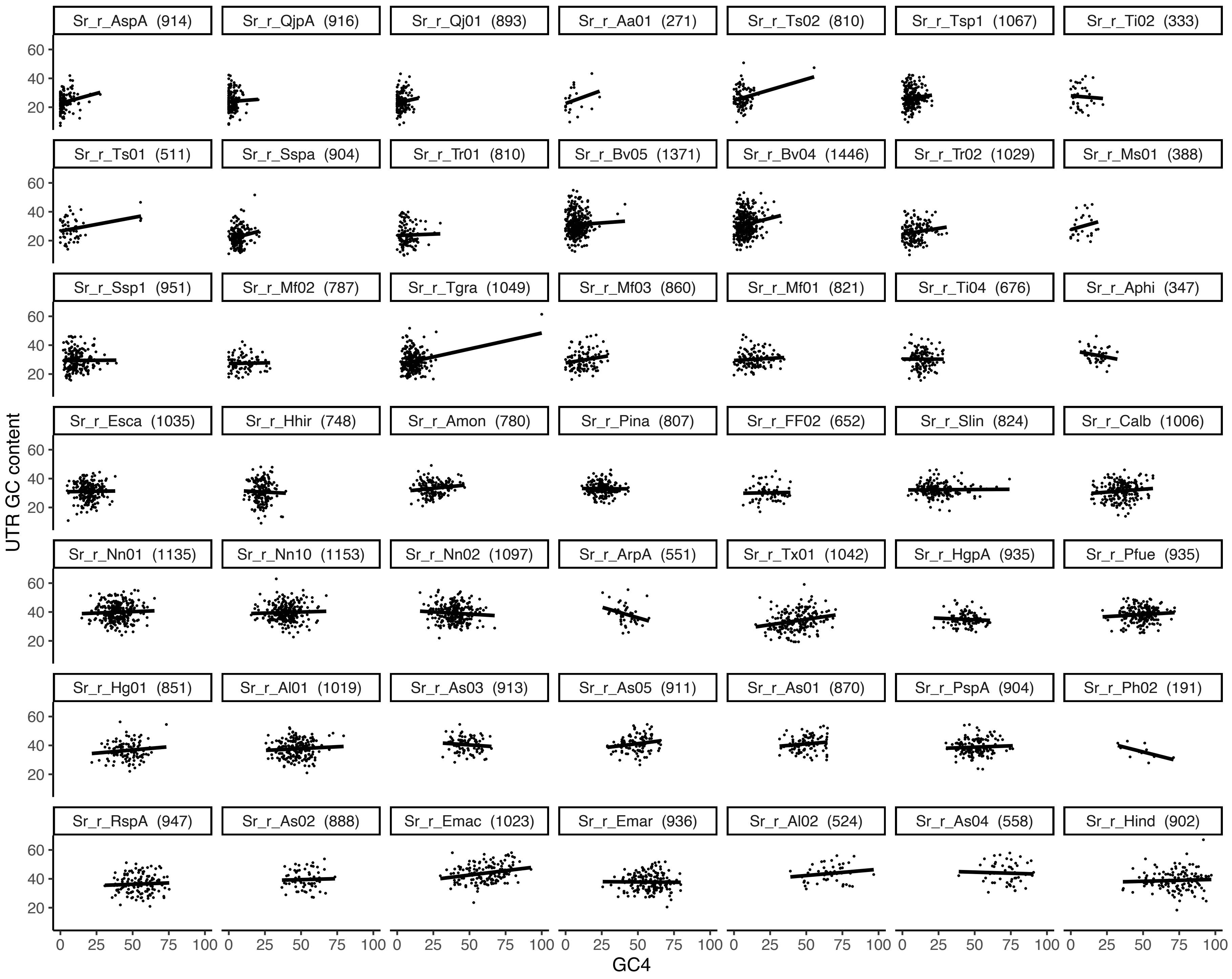
Sr_r_AspA (914)
Sr_r_QjpA (916)
Sr_r_Qj01 (893)
Sr_r_Aa01 (271)
Sr_r_Ts02 (810)
Sr_r_Tsp1 (1067)
Sr_r_Ti02 (333)
Sr_r_Ts01 (511)
Sr_r_Sspa (904)
Sr_r_Tr01 (810)
Sr_r_Bv05 (1371)
Sr_r_Bv04 (1446)
Sr_r_Tr02 (1029)
Sr_r_Ms01 (388)
Sr_r_Ssp1 (951)
Sr_r_Mf02 (787)
Sr_r_Tgra (1049)
Sr_r_Mf03 (860)
Sr_r_Mf01 (821)
Sr_r_Ti04 (676)
Sr_r_Aphi (347)
Sr_r_Esca (1035)
Sr_r_Hhir (748)
Sr_r_Amon (780)
Sr_r_Pina (807)
Sr_r_FF02 (652)
Sr_r_Slin (824)
Sr_r_Calb (1006)
Sr_r_Nn01 (1135)
Sr_r_Nn10 (1153)
Sr_r_Nn02 (1097)
Sr_r_ArpA (551)
Sr_r_Tx01 (1042)
Sr_r_HgpA (935)
Sr_r_Pfue (935)
Sr_r_Hg01 (851)
Sr_r_Al01 (1019)
Sr_r_As03 (913)
Sr_r_As05 (911)
Sr_r_As01 (870)
Sr_r_PspA (904)
Sr_r_Ph02 (191)
Sr_r_RspA (947)
Sr_r_As02 (888)
Sr_r_Emac (1023)
Sr_r_Emar (936)
Sr_r_Al02 (524)
Sr_r_As04 (558)
Sr_r_Hind (902)
UTR GC content
60
40
20
0
25
50
75
100
GC4

Fig. S5

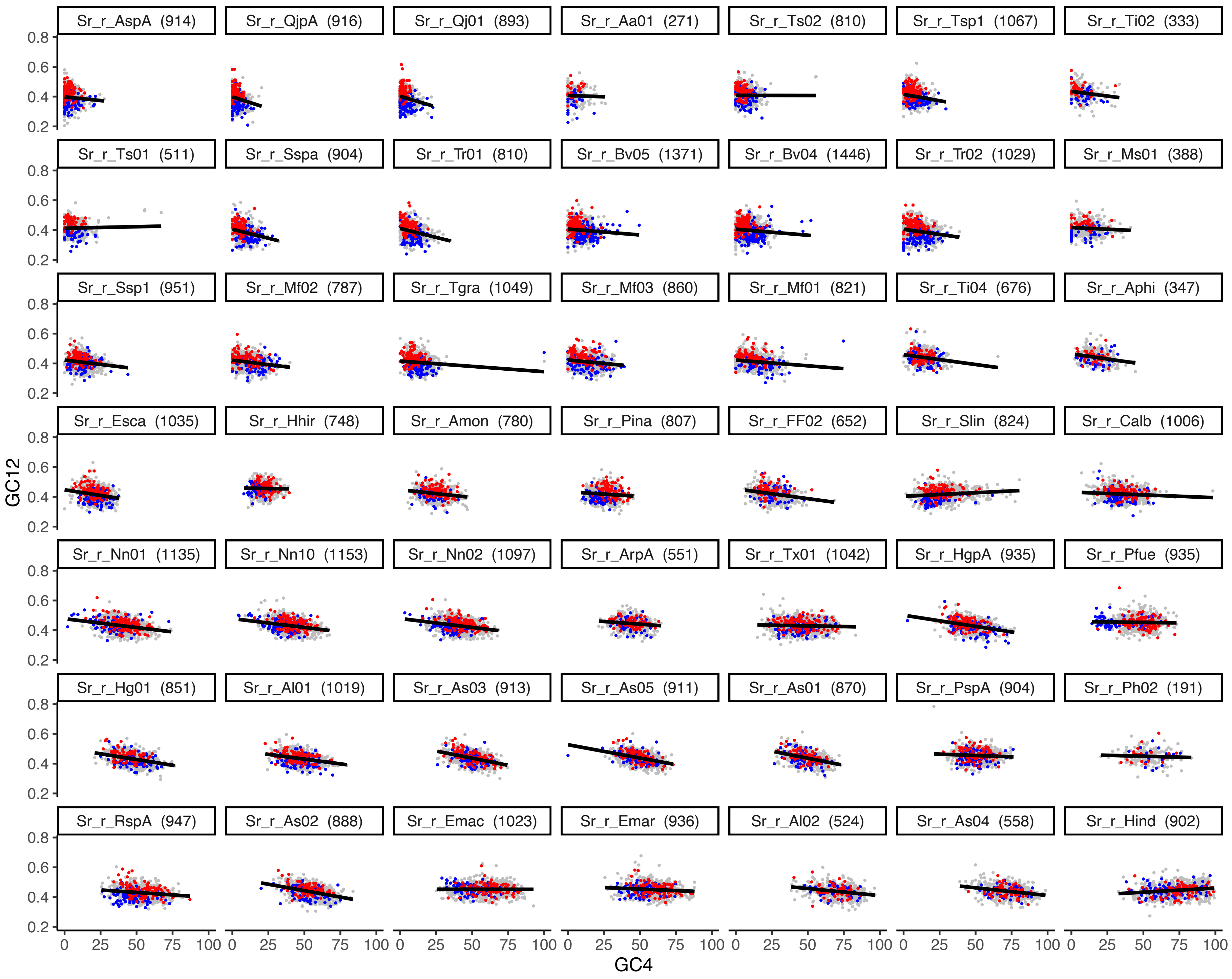

Sr_r_AspA (914)
Sr_r_QjpA (916)
Sr_r_Qj01 (893)
Sr_r_Aa01 (271)
Sr_r_Ts02 (810)
Sr_r_Tsp1 (1067)
Sr_r_Ti02 (333)
Sr_r_Ts01 (511)
Sr_r_Sspa (904)
Sr_r_Tr01 (810)
Sr_r_Bv05 (1371)
Sr_r_Bv04 (1446)
Sr_r_Tr02 (1029)
Sr_r_Ms01 (388)
Sr_r_Ssp1 (951)
Sr_r_Mf02 (787)
Sr_r_Tgra (1049)
Sr_r_Mf03 (860)
Sr_r_Mf01 (821)
Sr_r_Ti04 (676)
Sr_r_Aphi (347)
Sr_r_Esca (1035)
Sr_r_Hhir (748)
Sr_r_Amon (780)
Sr_r_Pina (807)
Sr_r_FF02 (652)
Sr_r_Slin (824)
Sr_r_Calb (1006)
Sr_r_Nn01 (1135)
Sr_r_Nn10 (1153)
Sr_r_Nn02 (1097)
Sr_r_ArpA (551)
Sr_r_Tx01 (1042)
Sr_r_HgpA (935)
Sr_r_Pfue (935)
Sr_r_Hg01 (851)
Sr_r_Al01 (1019)
Sr_r_As03 (913)
Sr_r_As05 (911)
Sr_r_As01 (870)
Sr_r_PspA (904)
Sr_r_Ph02 (191)
Sr_r_RspA (947)
Sr_r_As02 (888)
Sr_r_Emac (1023)
Sr_r_Emar (936)
Sr_r_Al02 (524)
Sr_r_As04 (558)
Sr_r_Hind (902)
GC12
0.2 0.4 0.6 0.8
GC4
0 25 50 75 100

Fig. S6

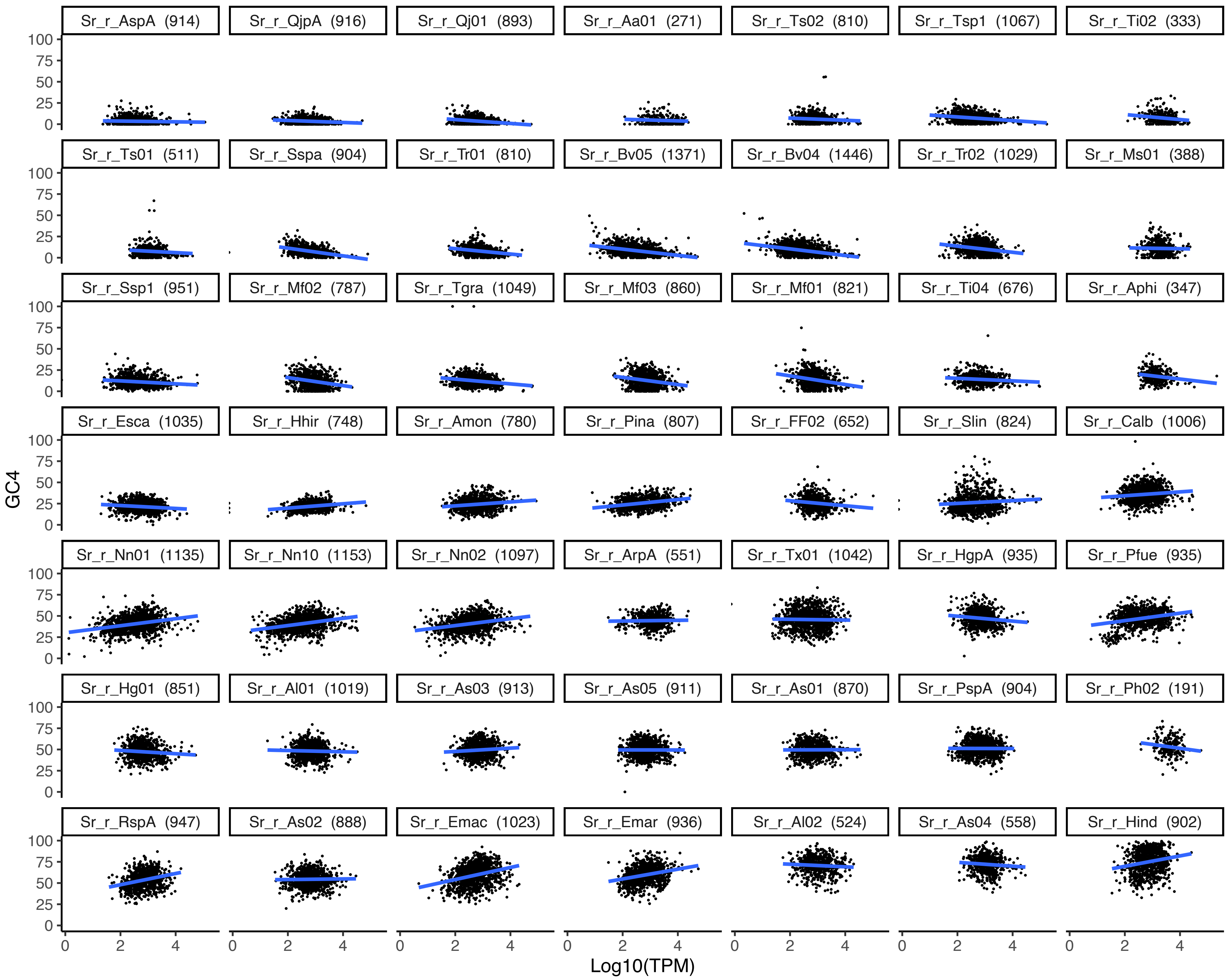

Sr_r_AspA (914)
Sr_r_QjpA (916)
Sr_r_Qj01 (893)
Sr_r_Aa01 (271)
Sr_r_Ts02 (810)
Sr_r_Tsp1 (1067)
Sr_r_Ti02 (333)
Sr_r_Ts01 (511)
Sr_r_Sspa (904)
Sr_r_Tr01 (810)
Sr_r_Bv05 (1371)
Sr_r_Bv04 (1446)
Sr_r_Tr02 (1029)
Sr_r_Ms01 (388)
Sr_r_Ssp1 (951)
Sr_r_Mf02 (787)
Sr_r_Tgra (1049)
Sr_r_Mf03 (860)
Sr_r_Mf01 (821)
Sr_r_Ti04 (676)
Sr_r_Aphi (347)
Sr_r_Esca (1035)
Sr_r_Hhir (748)
Sr_r_Amon (780)
Sr_r_Pina (807)
Sr_r_FF02 (652)
Sr_r_Slin (824)
Sr_r_Calb (1006)
Sr_r_Nn01 (1135)
Sr_r_Nn10 (1153)
Sr_r_Nn02 (1097)
Sr_r_ArpA (551)
Sr_r_Tx01 (1042)
Sr_r_HgpA (935)
Sr_r_Pfue (935)
Sr_r_Hg01 (851)
Sr_r_Al01 (1019)
Sr_r_As03 (913)
Sr_r_As05 (911)
Sr_r_As01 (870)
Sr_r_PspA (904)
Sr_r_Ph02 (191)
Sr_r_RspA (947)
Sr_r_As02 (888)
Sr_r_Emac (1023)
Sr_r_Emar (936)
Sr_r_Al02 (524)
Sr_r_As04 (558)
Sr_r_Hind (902)
GC4
0 25 50 75 100
0 2 4
Log10(TPM)

Fig. S7

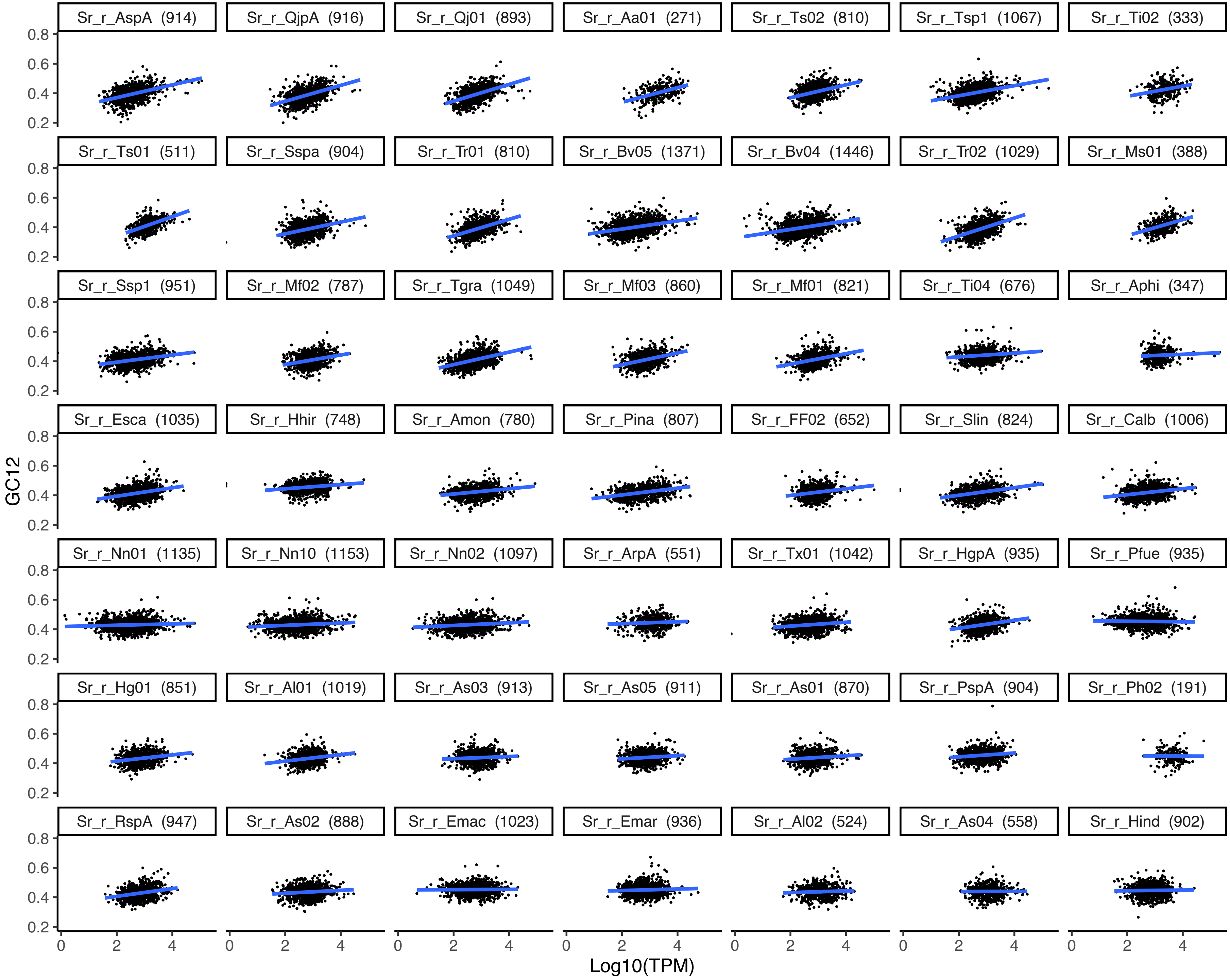

Sr_r_AspA (914)
Sr_r_QjpA (916)
Sr_r_Qj01 (893)
Sr_r_Aa01 (271)
Sr_r_Ts02 (810)
Sr_r_Tsp1 (1067)
Sr_r_Ti02 (333)
Sr_r_Ts01 (511)
Sr_r_Sspa (904)
Sr_r_Tr01 (810)
Sr_r_Bv05 (1371)
Sr_r_Bv04 (1446)
Sr_r_Tr02 (1029)
Sr_r_Ms01 (388)
Sr_r_Ssp1 (951)
Sr_r_Mf02 (787)
Sr_r_Tgra (1049)
Sr_r_Mf03 (860)
Sr_r_Mf01 (821)
Sr_r_Ti04 (676)
Sr_r_Aphi (347)
Sr_r_Esca (1035)
Sr_r_Hhir (748)
Sr_r_Amon (780)
Sr_r_Pina (807)
Sr_r_FF02 (652)
Sr_r_Slin (824)
Sr_r_Calb (1006)
Sr_r_Nn01 (1135)
Sr_r_Nn10 (1153)
Sr_r_Nn02 (1097)
Sr_r_ArpA (551)
Sr_r_Tx01 (1042)
Sr_r_HgpA (935)
Sr_r_Pfue (935)
Sr_r_Hg01 (851)
Sr_r_Al01 (1019)
Sr_r_As03 (913)
Sr_r_As05 (911)
Sr_r_As01 (870)
Sr_r_PspA (904)
Sr_r_Ph02 (191)
Sr_r_RspA (947)
Sr_r_As02 (888)
Sr_r_Emac (1023)
Sr_r_Emar (936)
Sr_r_Al02 (524)
Sr_r_As04 (558)
Sr_r_Hind (902)
GC12
0.8
0.6
0.4
0.2
0
2
4
Log10(TPM)

Fig. S8

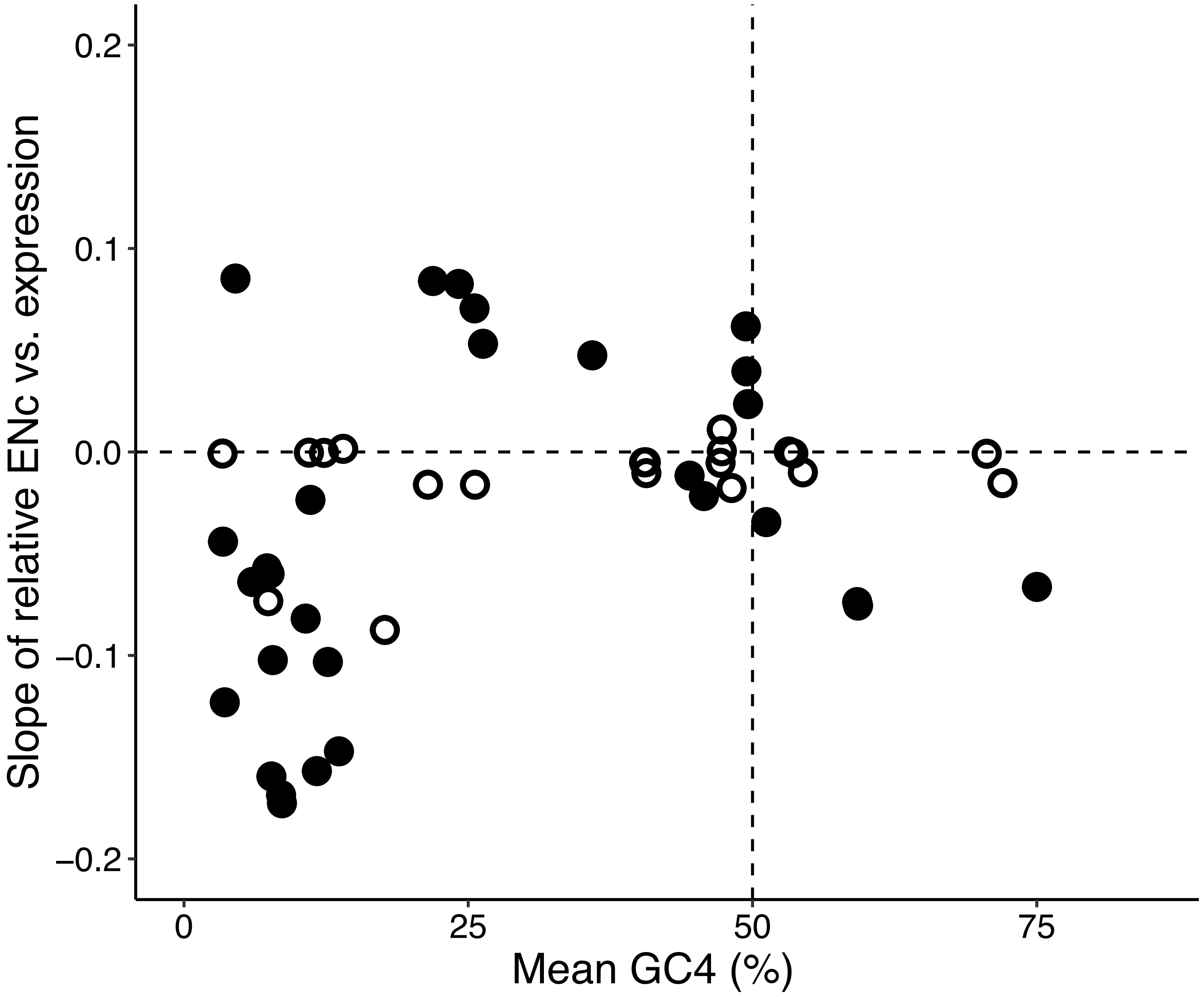
Slope of relative ENc vs. expression
0.2
0.1
0.0
−0.1
−0.2
0
25
50
75
Mean GC4 (%)

Fig S9

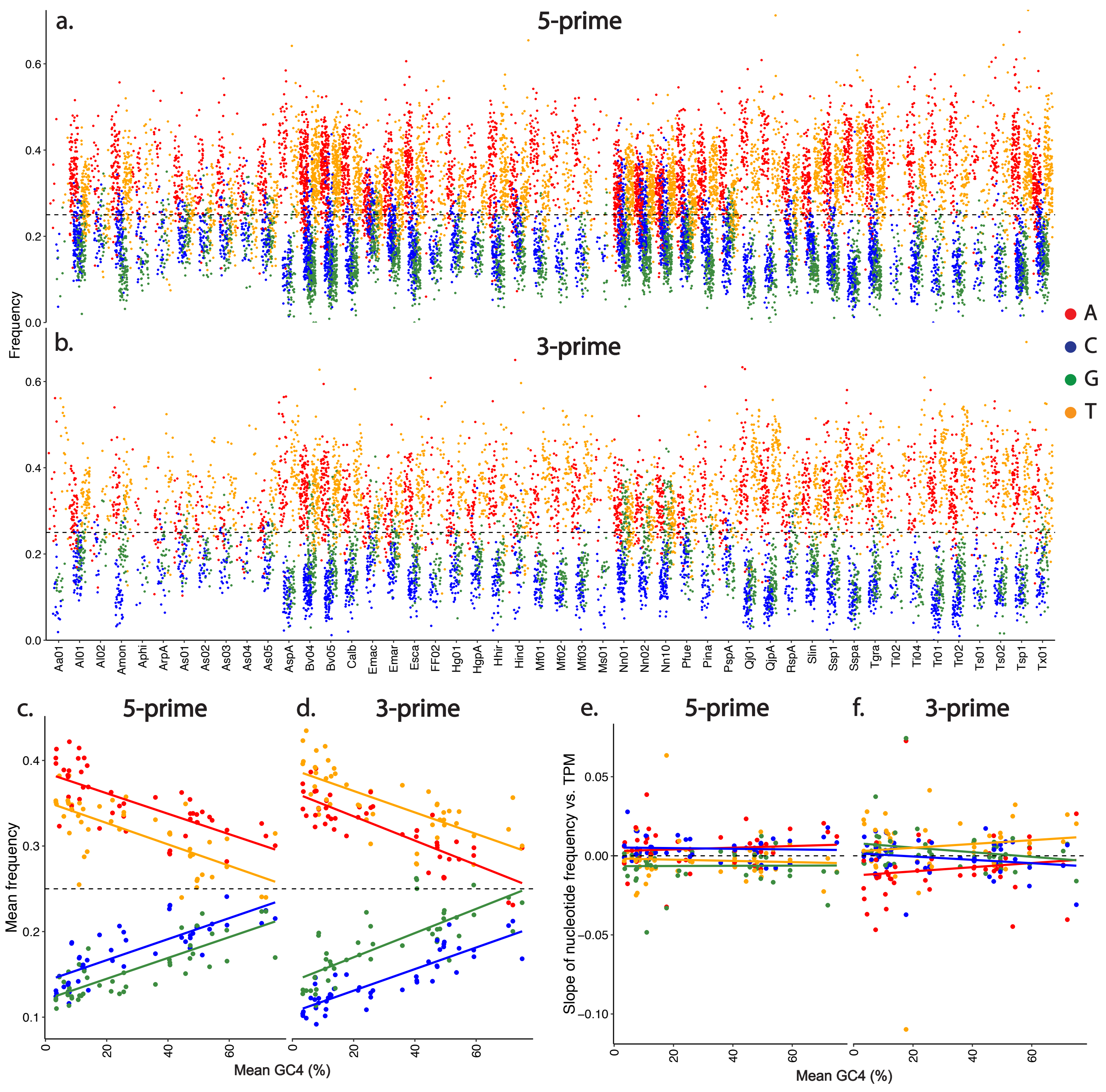

a.
5-prime
b.
3-prime
Frequency
0.0
0.2
0.4
0.6
A
C
G
T
Aa01
Al01
Al02
Amon
Aphi
ArpA
As01
As02
As03
As04
As05
AspA
Bv04
Bv05
Calb
Emac
Emar
Esca
FF02
Hg01
HgpA
Hhir
Hind
Mf01
Mf02
Mf03
Ms01
Nn01
Nn02
Nn10
Pfue
Pina
PspA
Qj01
QjpA
RspA
Slin
Ssp1
Sspa
Tgra
Ti02
Ti04
Tr01
Tr02
Ts01
Ts02
Tsp1
Tx01
c.
5-prime
d.
3-prime
Mean frequency
0.1
0.2
0.3
0.4
0
20
40
60
Mean GC4 (%)
e.
5-prime
f.
3-prime
Slope of nucleotide frequency vs. TPM
−0.10
−0.05
0.00
0.05
Mean GC4 (%)

Fig. S10

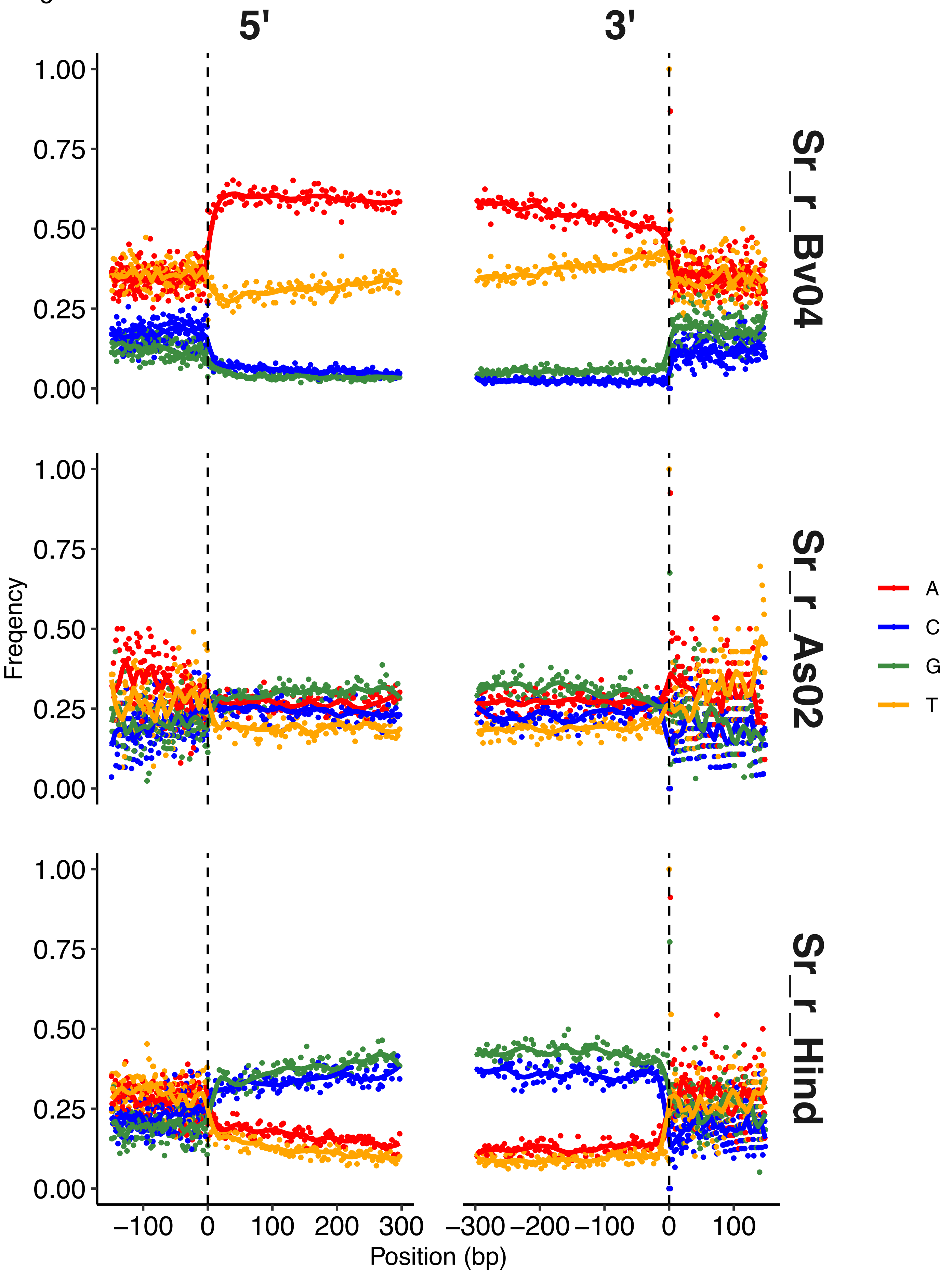

5'
3'
Sr_r_Bv04
Sr_r_As02
Sr_r_Hind
Freqency
Position (bp)
1.00
0.75
0.50
0.25
0.00
−100
0
100
200
300
−300
−200
−100
A
C
G
T

Fig. S11

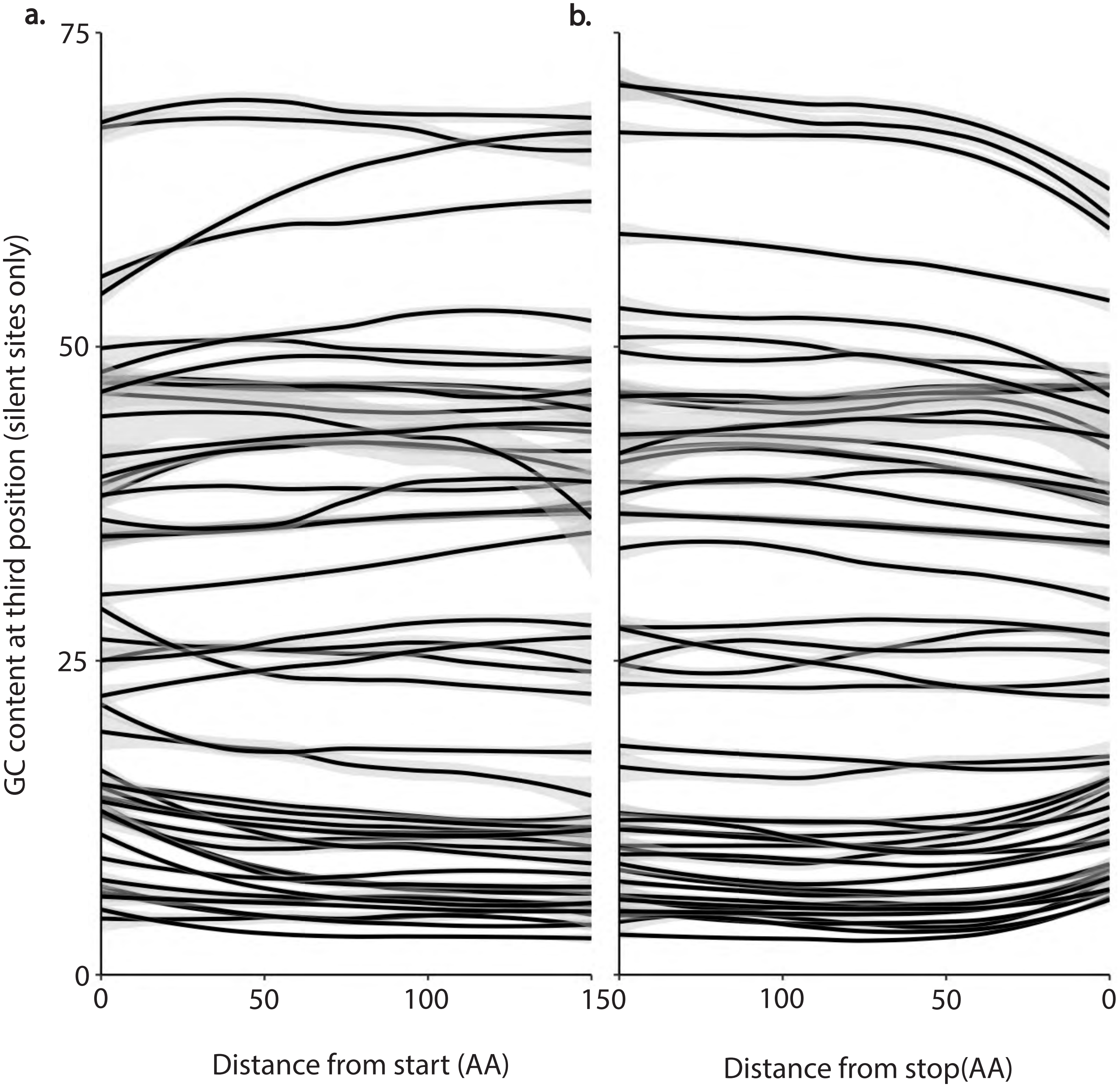

a.
b.
75
50
25
0
GC content at third position (silent sites only)
0
50
100
150
Distance from start (AA)
100
50
0
Distance from stop(AA)

Fig. S12

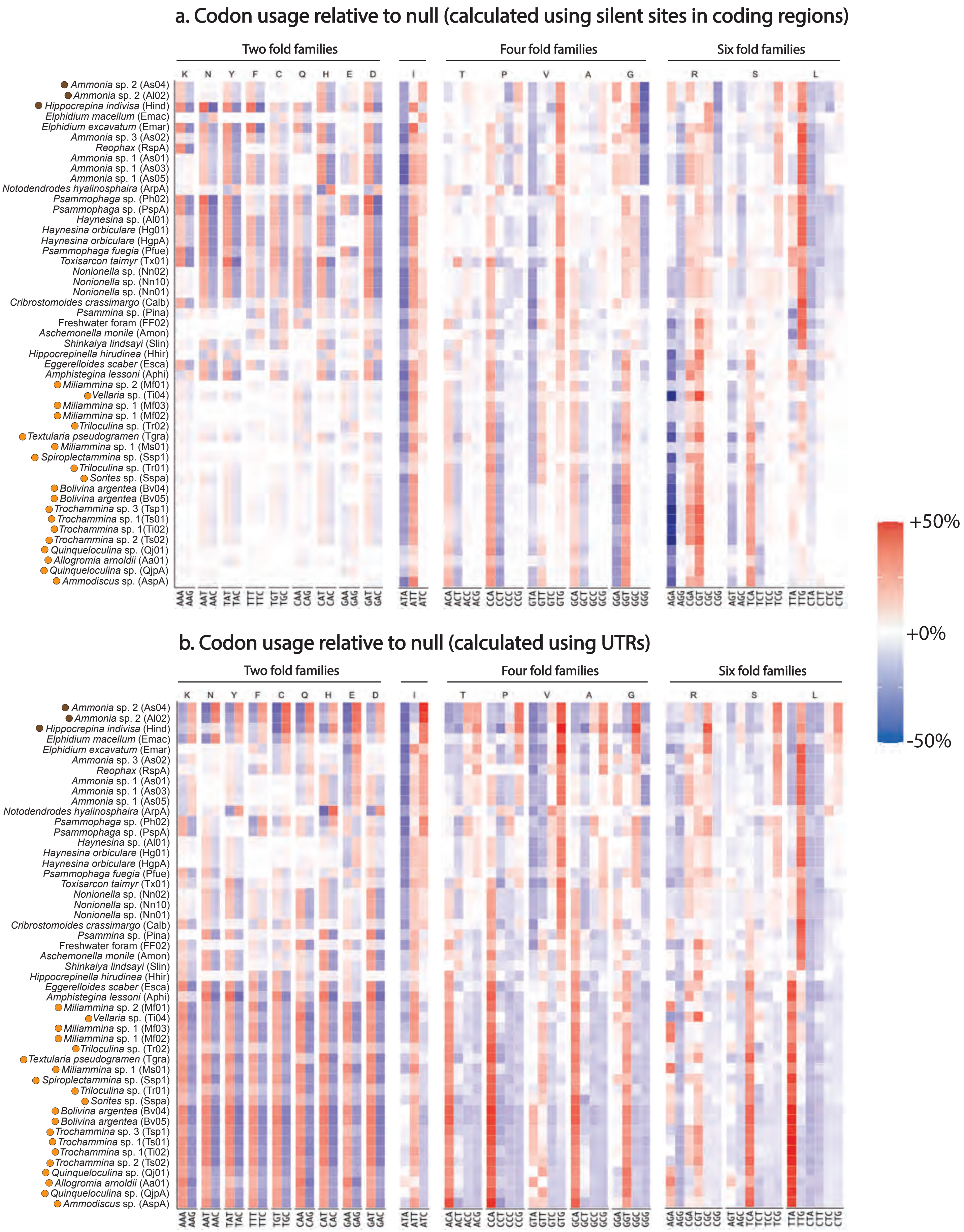

a. Codon usage relative to null (calculated using silent sites in coding regions)
b. Codon usage relative to null (calculated using UTRs)
Two fold families
Four fold families
Six fold families
K N Y F C Q H E D
I
T P V A G
R S L
Ammonia sp. 2 (As04)
Ammonia sp. 2 (Al02)
Hippocrepina indivisa (Hind)
Elphidium macellum (Emac)
Elphidium excavatum (Emar)
Ammonia sp. 3 (As02)
Reophax (RspA)
Ammonia sp. 1 (As01)
Ammonia sp. 1 (As03)
Ammonia sp. 1 (As05)
Notodendrodes hyalinosphaira (ArpA)
Psammophaga sp. (Ph02)
Psammophaga sp. (PspA)
Haynesina sp. (Al01)
Haynesina orbiculare (Hg01)
Haynesina orbiculare (HgpA)
Psammophaga fuegia (Pfue)
Toxisarcon taimyr (Tx01)
Nonionella sp. (Nn02)
Nonionella sp. (Nn10)
Nonionella sp. (Nn01)
Cribrostomoides crassimargo (Calb)
Psammina sp. (Pina)
Freshwater foram (FF02)
Aschemonella monile (Amon)
Shinkaiya lindsayi (Slin)
Hippocrepinella hirudinea (Hhir)
Eggerelloides scaber (Esca)
Amphistegina lessoni (Aphi)
Miliammina sp. 2 (Mf01)
Vellaria sp. (Ti04)
Miliammina sp. 1 (Mf03)
Miliammina sp. 1 (Mf02)
Triloculina sp. (Tr02)
Textularia pseudogramen (Tgra)
Miliammina sp. 1 (Ms01)
Spiroplectammina sp. (Ssp1)
Triloculina sp. (Tr01)
Sorites sp. (Sspa)
Bolivina argentea (Bv04)
Bolivina argentea (Bv05)
Trochammina sp. 3 (Tsp1)
Trochammina sp. 1(Ts01)
Trochammina sp. 1(Ti02)
Trochammina sp. 2 (Ts02)
Quinqueloculina sp. (Qj01)
Allogromia arnoldii (Aa01)
Quinqueloculina sp. (QjpA)
Ammodiscus sp. (AspA)
AAA AAG AAT AAC TAT TAC TTT TTC TGT TGC CAA CAG CAT CAC GAA GAG GAT GAC
ATA ATT ATC
ACA ACT ACC ACG CCA CCT CCC CCG GTA GTT GTC GTG GCA GCT GCC GCG GGA GGT GGC GGG
AGA AGG CGA CGT CGC CGG AGT AGC TCA TCT TCC TCG TTA TTG CTA CTT CTC CTG
+50%
+0%
-50%

Fig. S13

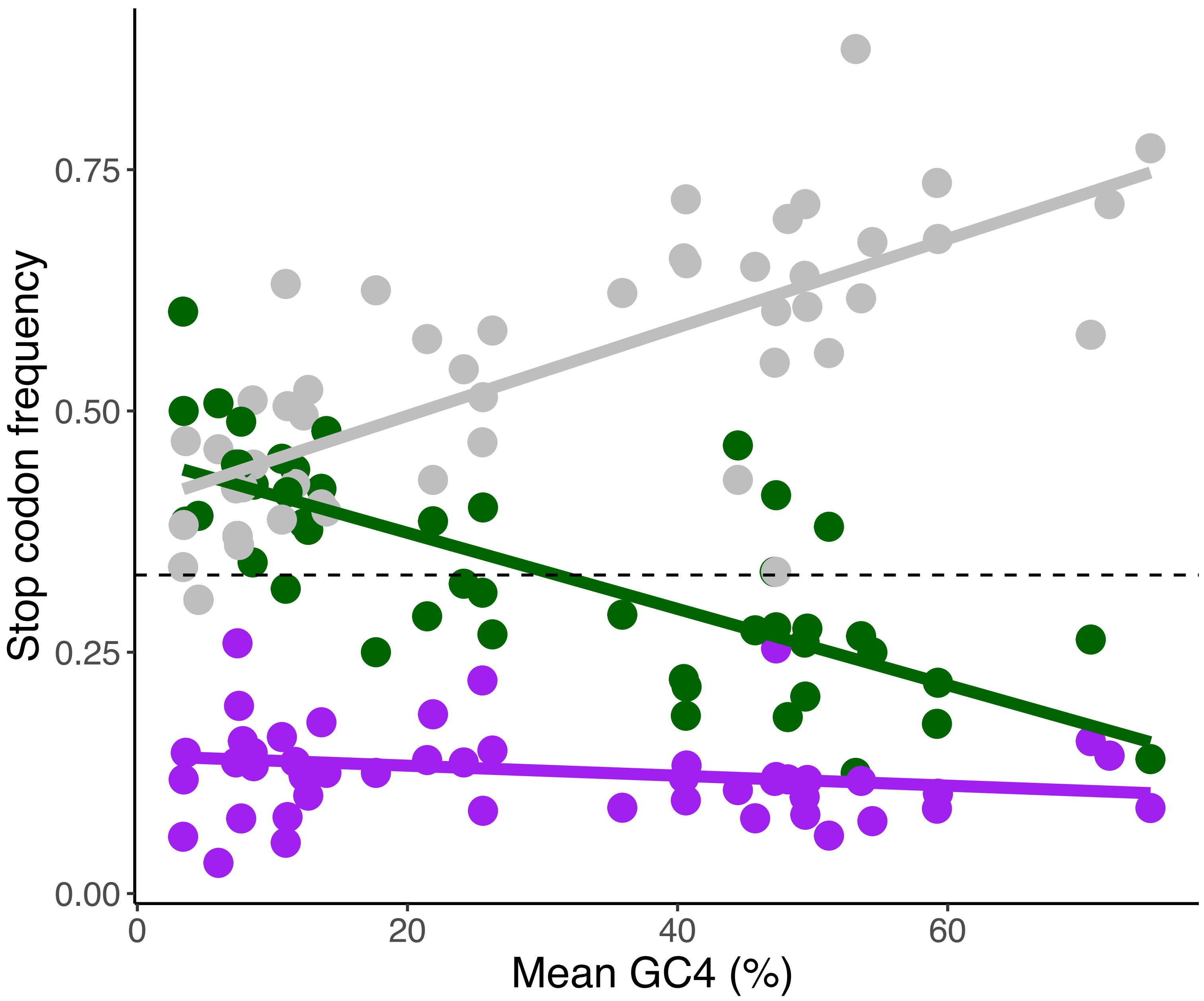

Fig. S14

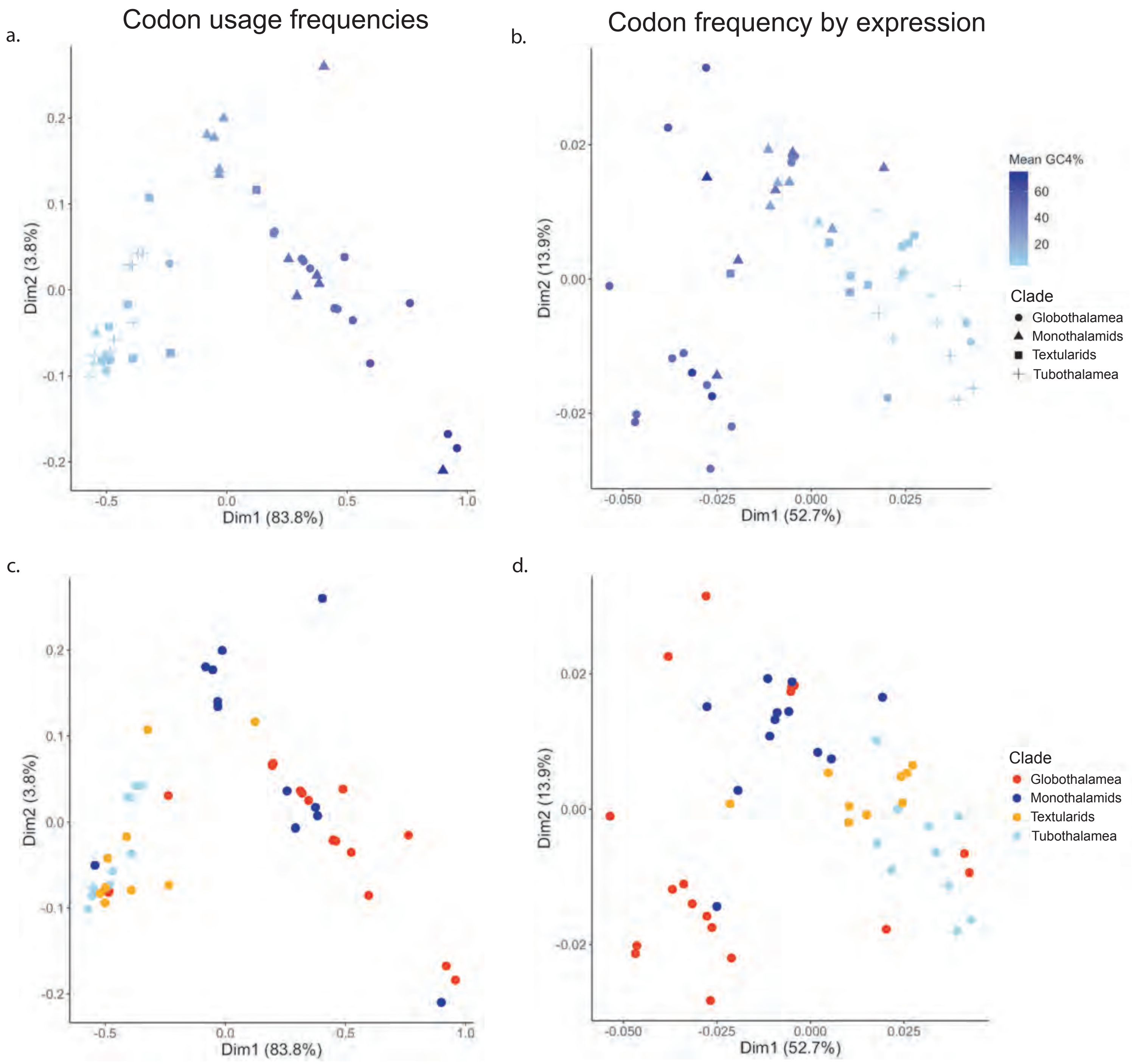

a.
Codon usage frequencies
Dim2 (3.8%)
Dim1 (83.8%)
0.2
0.1
0.0
-0.1
-0.2
-0.5
0.0
0.5
1.0
b.
Codon frequency by expression
Dim2 (13.9%)
Dim1 (52.7%)
0.02
0.00
-0.02
-0.050
-0.025
0.000
0.025
Mean GC4%
60
40
20
Clade
Globothalamea
Monothalamids
Textularids
Tubothalamea
c.
Dim2 (3.8%)
Dim1 (83.8%)
d.
Dim2 (13.9%)
Dim1 (52.7%)
Clade
Globothalamea
Monothalamids
Textularids
Tubothalamea

Fig. S15

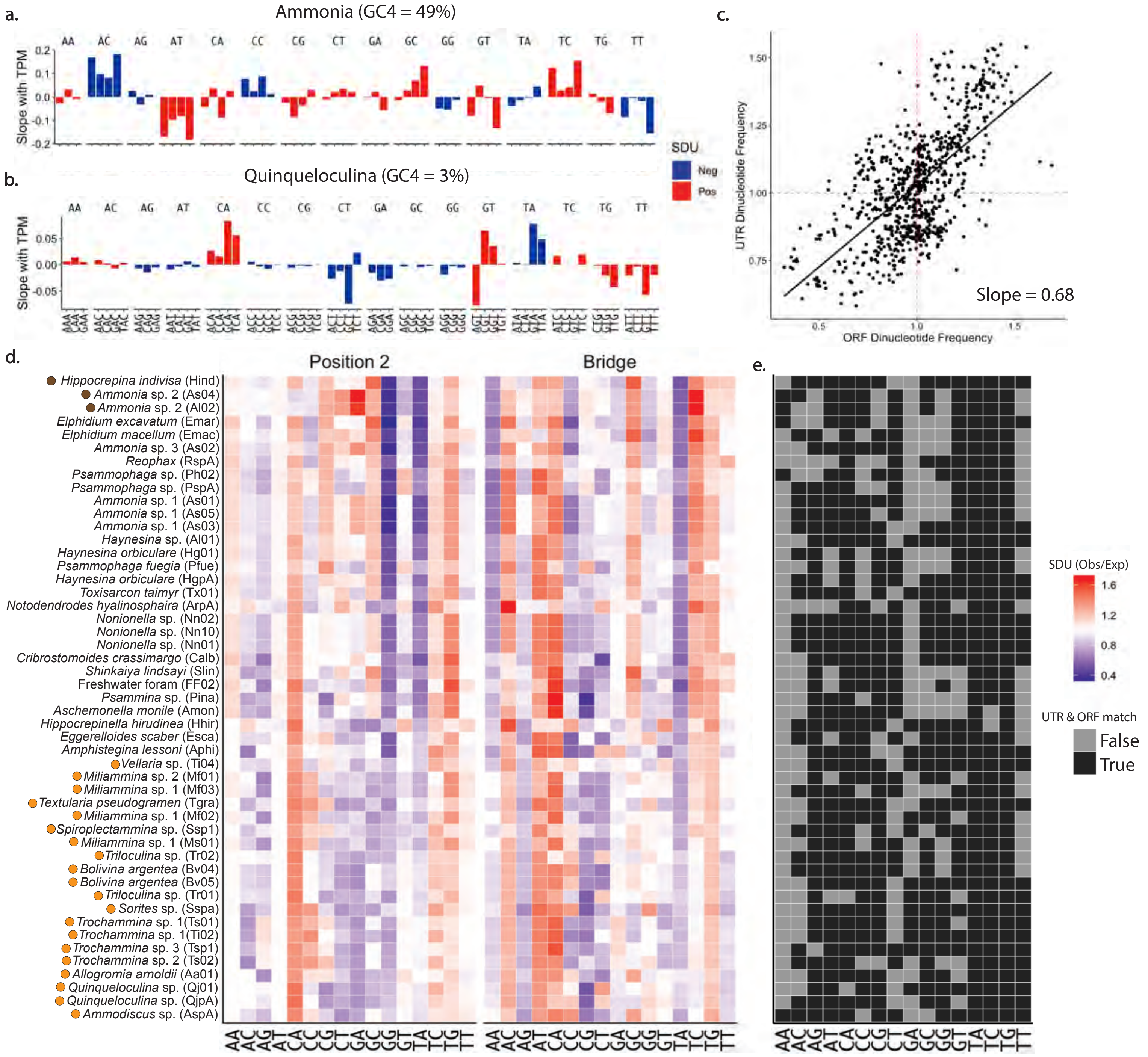
a.
Ammonia (GC4 = 49%)
Slope with TPM
b.
Quinqueloculina (GC4 = 3%)
SDU
Neg
Pos
c.
UTR Dinucleotide Frequency
ORF Dinucleotide Frequency
Slope = 0.68
d.
Position 2
Bridge
Hippocrepina indivisa (Hind)
Ammonia sp. 2 (As04)
Ammonia sp. 2 (Al02)
Elphidium excavatum (Emar)
Elphidium macellum (Emac)
Ammonia sp. 3 (As02)
Reophax (RspA)
Psammophaga sp. (Ph02)
Psammophaga sp. (PspA)
Ammonia sp. 1 (As01)
Ammonia sp. 1 (As05)
Ammonia sp. 1 (As03)
Haynesina sp. (Al01)
Haynesina orbiculare (Hg01)
Psammophaga fuegia (Pfue)
Haynesina orbiculare (HgpA)
Toxisarcon taimyr (Tx01)
Notodendrodes hyalinosphaira (ArpA)
Nonionella sp. (Nn02)
Nonionella sp. (Nn10)
Nonionella sp. (Nn01)
Cribrostomoides crassimargo (Calb)
Shinkaiya lindsayi (Slin)
Freshwater foram (FF02)
Psammina sp. (Pina)
Aschemonella monile (Amon)
Hippocrepinella hirudinea (Hhir)
Eggerelloides scaber (Esca)
Amphistegina lessoni (Aphi)
Vellaria sp. (Ti04)
Miliammina sp. 2 (Mf01)
Miliammina sp. 1 (Mf03)
Textularia pseudogramen (Tgra)
Miliammina sp. 1 (Mf02)
Spiroplectammina sp. (Ssp1)
Miliammina sp. 1 (Ms01)
Triloculina sp. (Tr02)
Bolivina argentea (Bv04)
Bolivina argentea (Bv05)
Triloculina sp. (Tr01)
Sorites sp. (Sspa)
Trochammina sp. 1(Ts01)
Trochammina sp. 1(Ti02)
Trochammina sp. 3 (Tsp1)
Trochammina sp. 2 (Ts02)
Allogromia arnoldii (Aa01)
Quinqueloculina sp. (Qj01)
Quinqueloculina sp. (QjpA)
Ammodiscus sp. (AspA)
e.
SDU (Obs/Exp)
UTR & ORF match
False
True

Fig. S16

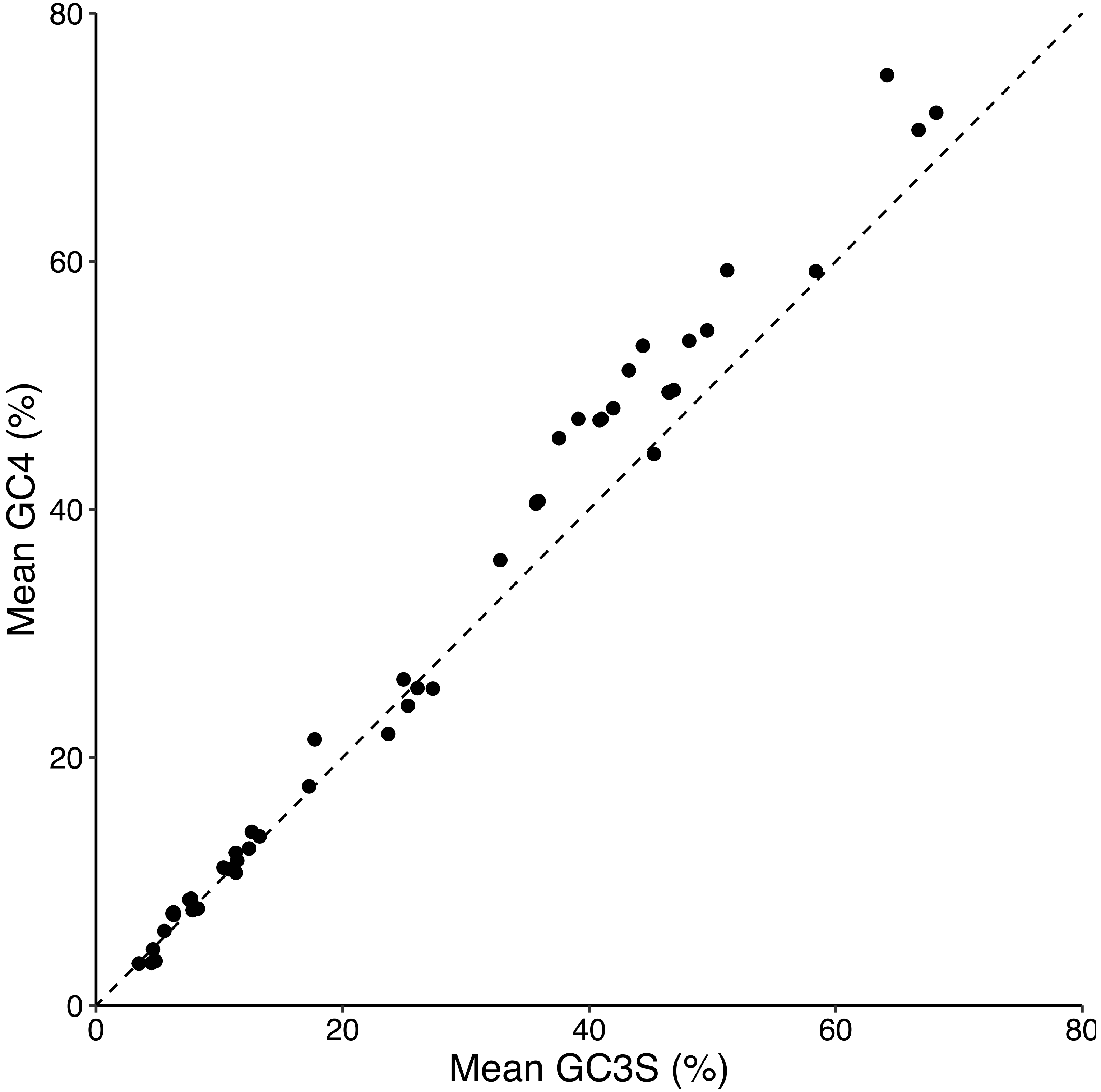

Fig. S17

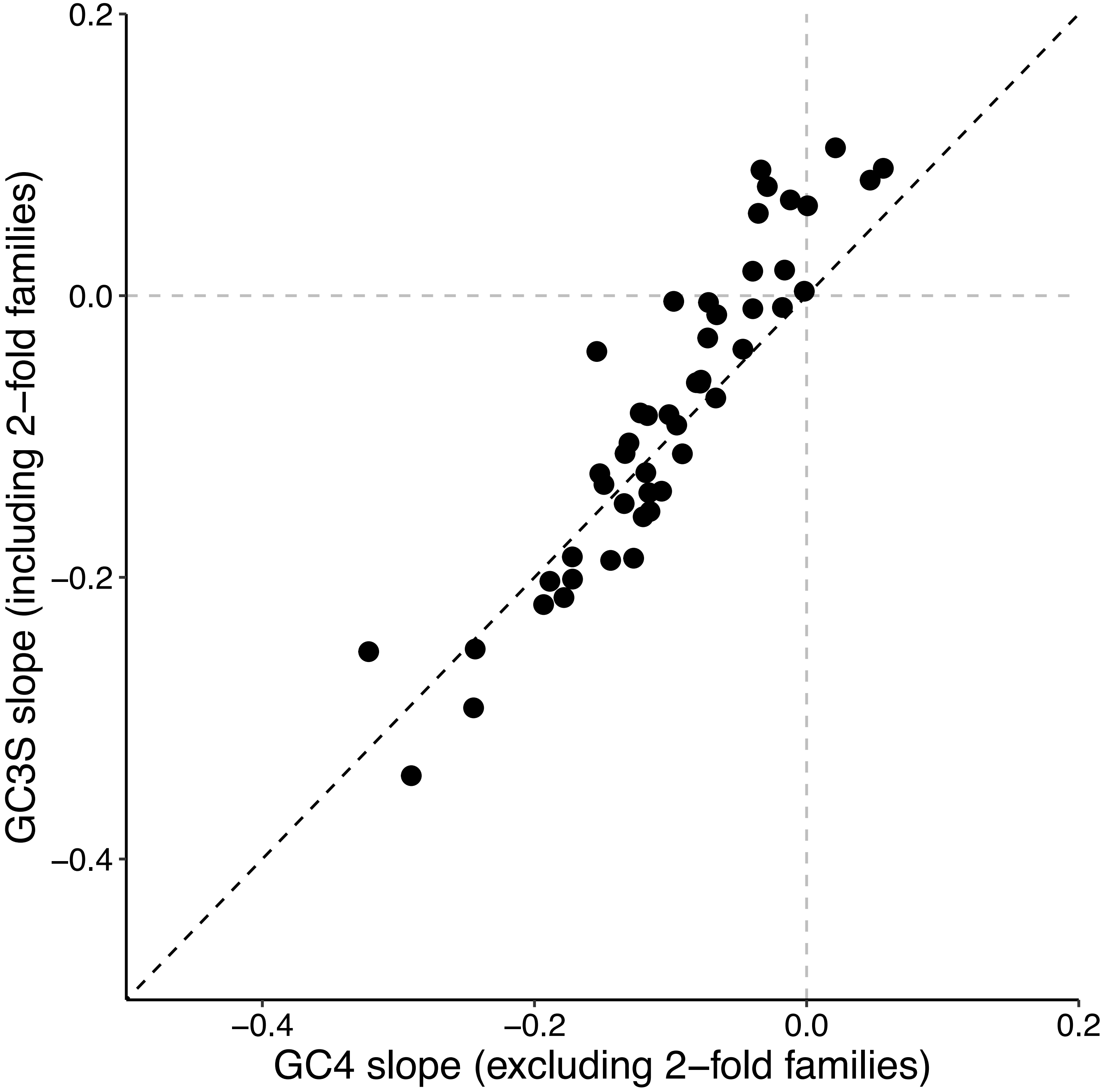

0.2
0.0
−0.2
−0.4
GC3S slope (including 2−fold families)
−0.4
−0.2
0.0
0.2
GC4 slope (excluding 2−fold families)
